# Naphthalene Metabolites From Long‐Term Environmental Tobacco Smoke Induce the Aging of Retinal Pigment Epithelium

**DOI:** 10.1111/acel.70150

**Published:** 2025-06-20

**Authors:** Tingting Cui, Qingjian Ou, Zhe Wang, Ye Zhou, Jinyuan Xu, Yifan Liu, Yanlong Bi, Xiaoliang Jin, Jie Chen, Furong Gao, Juan Wang, Jieping Zhang, Lixia Lu, Guo‐Tong Xu, Caixia Jin, Haibin Tian, Jing‐Ying Xu

**Affiliations:** ^1^ Department of Ophthalmology and Laboratory of Clinical and Visual Sciences of Tongji Eye Institute, Tongji Hospital, School of Medicine Tongji University Shanghai China; ^2^ Department of Physiology, College of Basic Medical Sciences Naval Medical University Shanghai China; ^3^ Department of Ophthalmology, Ninth People's Hospital Shanghai Jiao Tong University School of Medicine Shanghai China

**Keywords:** 1,2‐Dihydroxynaphthalene, 1,2‐Naphthoquinone, cigarette exposure, environmental tobacco smoke, RPE senescence, smoking cessation

## Abstract

Tobacco use is the main source of indoor air pollution and contains a variety of toxic components. The smoke from burning cigarettes is a key environmental risk factor that leads to accelerated aging and the occurrence of numerous diseases. Meanwhile, cigarette smoke and aging are both prominent risk factors for age‐related macular degeneration (AMD). This study demonstrates that long‐term exposure to cigarette smoke can impair retinal function and induce the aging of retinal pigment epithelium (RPE). Meanwhile, the plasma of rats after long‐term exposure to cigarette smoke can trigger DNA damage and cellular senescence in vitro. In addition, naphthalene and its metabolites (1,2‐dihydroxynaphthalene and 1,2‐naphthoquinone) derived from cigarette smoke have been identified as an important factor contributing to RPE damage caused by cigarette exposure. Finally, we found that the aging of RPE induced by smoking can be alleviated through smoking cessation, probably because quitting smoking reduces the accumulation of these toxic chemicals in plasma and within the eyes.

## Graphical abstract

Cigarette exposure can induce the senescence of retinal pigment epithelium (RPE). The accumulation of naphthalene metabolites, namely 1,2‐dihydronaphthalene and 1,2‐naphthoquinone, in rats is a key factor in this process. In ARPE‐19 cells, the plasma of rats exposed to cigarette smoke can cause a significant increase in the level of reactive oxygen species (ROS). In the case of short‐term exposure, related molecules such as γH2Ax, LaminB1, P16, and senescence—associated secretory phenotype (SASP) change, thus inducing cellular senescence. As the exposure time prolongs, the AMPK/SIRT3 signaling pathway is affected, which then acts on P53/P21, further exacerbating the process of cellular senescence. Notably, the plasma of rats after smoking cessation can improve the cellular senescence state in in vitro experiments. This phenomenon is most likely attributed to the fact that smoking cessation reduces the accumulation of naphthalene metabolites (1,2‐dihydronaphthalene and 1,2‐naphthoquinone) in rats.

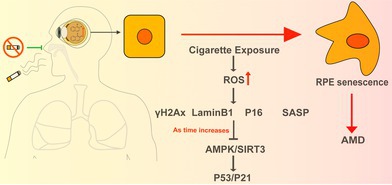

## 1 Introduction

Smoking is an addictive behavior and the most common form of tobacco use worldwide. However, while people enjoy the so‐called “sense of relaxation” from smoking, they often overlook the serious hazards of environmental tobacco smoke (ETS). Currently, among the many factors affecting indoor air quality, ETS has emerged as a primary source of pollution. Tobacco smoke contains over 6000 identified chemical components, including 80 known carcinogens (Karimi et al. 2023; Li and Hecht 2022; Wang et al. 2024). These chemicals are absorbed through the lungs into the bloodstream and distributed throughout the body (Kim et al. 2023).

Age‐related macular degeneration (AMD), the leading cause of severe, incurable vision loss in people over 55, affects approximately 196 million individuals worldwide (Cabral de Guimaraes et al. 2022; Fleckenstein et al. 2024). AMD is characterized by aging and loss of retinal pigment epithelium (RPE), and progressive pathological changes, including degeneration of the photoreceptor‐RPE complex (Deng et al. 2022; Kaufmann and Han 2024). RPE, which plays a key role in maintaining photoreceptor function, undergoes multiple age‐related changes. These include melanin loss, drusen formation, Bruch's membrane thickening, increased residual body density, altered basal folds, lipofuscin accumulation, basal deposit formation on/in Bruch's membrane, and microvillar atrophy. Consequently, the RPE becomes unable to sustain critical retinal homeostasis functions—including nutrient transport to photoreceptors and metabolic waste removal (Blasiak et al. 2022; Ma et al. 2023). Alongside age and genetic predisposition, smoking ranks among the strongest established environmental risk factors for AMD (Nakanishi et al. 2010). The thickness of the nerve fiber layer around the macula, choroid, and optic disc is significantly reduced in smokers compared to non‐smokers, with the thinning being more pronounced in long‐term smokers (Belmouhand et al. 2022; Woodell and Rohrer 2014; Yang, Song, et al. 2022). Epidemiological evidence further demonstrates that smoking induces earlier AMD onset and accelerates disease progression relative to non‐smokers, with smokers facing a 2‐ to 4‐fold higher AMD risk (Abusharkh et al. 2023; Istrate et al. 2021; Kuan et al. 2021; Thornton et al. 2005). Experimental evidence further indicates that long‐term cigarette smoke exposure induces early AMD pathology in mice, characterized by pronounced RPE damage. This supports that smoking accelerates AMD progression by disrupting retinal pigment epithelial function (Feng et al. 2022; Fujihara et al. 2008; Hanus et al. 2015; Ma et al. 2023; Wang, Kaya, et al. 2020).

In this study, we developed an experimental cigarette smoke exposure model for rats to investigate the effects of harmful smoke components on the retinal pigment epithelium (RPE) and their underlying cellular mechanisms in AMD pathogenesis. The primary toxic metabolites in cigarette smoking were identified. Furthermore, we found that the imperative of cessation from smoking can mitigate the toxicity towards RPE cells, which slows cellular senescence and minimizes retinal impairment. This work provides novel insights into cigarette smoke's pathological effects on ocular health and broader clinical implications for human disease prevention.

## 2 Results

### 2.1 Long‐Term Cigarette Exposure Caused RPE Aging

**FIGURE 1:**
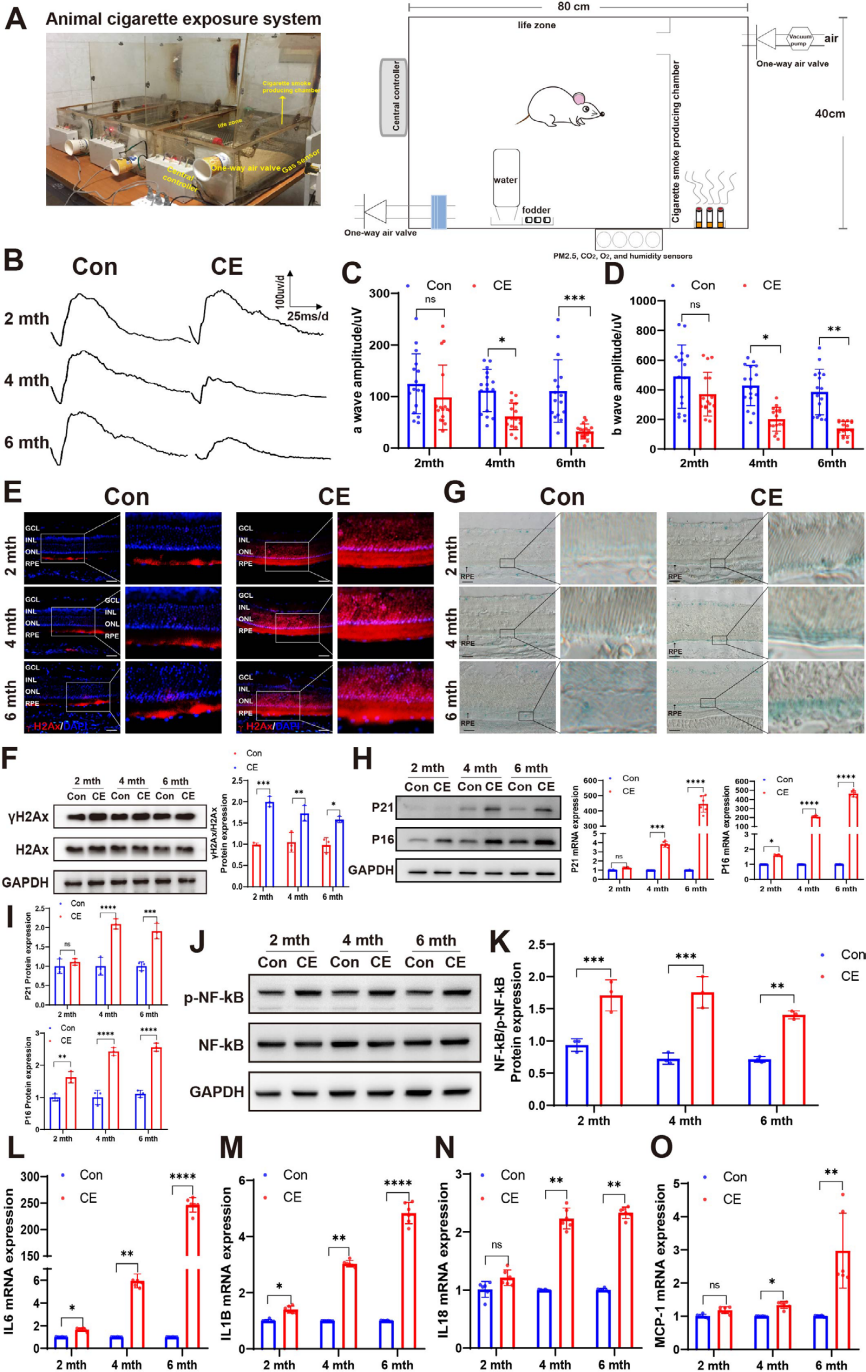
Cigarette exposure decreased the visual function and induced the senescence of RPE in rats. (A) Design and actual working drawing of cigarette exposure model box. (B) f‐ERG waves recorded from normal air‐exposed rat group (Con) and cigarette‐exposed rat group (CE). (Light intensity: 6.325 × 10^−2^) (C) Quantitative analysis of a‐wave amplitude in (B). *n* = 8. (D) Quantitative analysis of b‐wave amplitude in (B). *n* = 8. (E) Representative images of γH2Ax in the retina, γH2Ax (red) and nuclear DNA stained with DAPI (blue). Scale bar is 20 μm; *n* = 3. (F) Western blot analysis and quantification of γH2Ax and H2Ax expression in RBCC. *n* = 3. (G) SA‐β‐Gal staining in retina. Scale bar is 20 μm; *n* = 3. (H) Western blot analysis and quantification of P21 and P16 expression in RBCC. *n* = 3. (I) The expression of P21 and P16 in RBCC was detected with qPCR. *n* = 3. (J) Western blot analysis of p‐NF‐κB and NF‐κB expression in RBCC. *n* = 3. (K) Quantitative analysis of protein expression levels in (J). *n* = 3. (L) The expression IL‐6, IL‐1B (M), IL‐18 (N) and MCP‐1 (O) in RBCC was detected with qPCR. *n* = 3. *p* value measured by one‐way ANOVA and post hoc Bonferroni's test. Data are presented as means ± SD. Ns, not significant, *p < 0.05; **p < 0.01; ***p < 0.001; ****p < 0.0001.

A rat model was established using a custom‐designed cigarette exposure system to simulate prolonged smoking cigarette exposure on the retina toxicity. As shown in Figure 1A, it contains a cigarette exposure room and the living room with air sensors and a controller to mimic the process of normal smoking. The real‐time concentrations of O_2_ and CO_2_, which were recorded by the sensors, were lower than the minimum toxic dosage (Figure S1). The flash electroretinography (f‐ERG) was involved to evaluate the visual function of the animals. After 2 months of exposure, no significant differences in a‐wave and b‐wave responses were observed between control and cigarette‐exposed rats. With continuous exposure time, the a‐wave and b‐wave amplitudes in rats exposed for 4 months were significantly reduced compared to those at 2 months and further decreased at 6 months (Figure 1B–D). These findings indicate that prolonged cigarette exposure leads to diminished retinal function in this rat model. DNA damage, marked by γH2Ax expression, increased in the outer nuclear layer (ONL) and RPE after 2 months of cigarette exposure (Figure 1E,F). Subsequently, through senescence staining, it was found that a small number of positive senescence staining results began to appear in the RPE layer of rats after 2 months of cigarette exposure. When the exposure time was extended to 4 months, the RPE layer of rats exhibited extremely significant senescence staining characteristics. As the exposure time prolonged, the senescence staining became even more prominent. This result is consistent with the decline in retinal function. Specifically, prolonged cigarette exposure time increased the number of senescent RPE cells, leading to a notable decline in retinal function (Figure 1G). This strongly suggests that RPE cells are exquisitely sensitive to cigarette exposure.

Then, we isolated the RPE—Bruch's membrane—Choroid Complex (RBCC) to detect the changes in the senescence markers P16 and P21 at the protein and mRNA levels following cigarette exposure. P16 expression increased following 2 months of cigarette smoke exposure, whereas P21 levels rose after 4 months (Figure 1H,I). Besides, senescent cells can develop a characteristic pathogenic senescence‐associated secretory phenotype (SASP) that drives secondary senescence, disrupts tissue homeostasis, and leads to loss of tissue repair and regeneration (Yun et al. 2023; Zhang et al. 2022). NF‐κB phosphorylation, a key activation step for this main SASP mediator, was increased by 2 months of cigarette exposure in the RBCC (Figure 1J,K). Among SASP, mRNA levels of IL‐6 and IL‐1B in RBCC of cigarette‐exposed rats for 2 months were increased. At 4 months, the SASP (IL‐6, IL‐1B, IL‐18, and MCP‐1) expression in the cigarette exposure group was significantly higher than that in the control group (Figure 1L–O). These results suggest that long‐term (> 2 months) cigarette exposure induces RPE senescence and visual degradation in rats.

**FIGURE 2:**
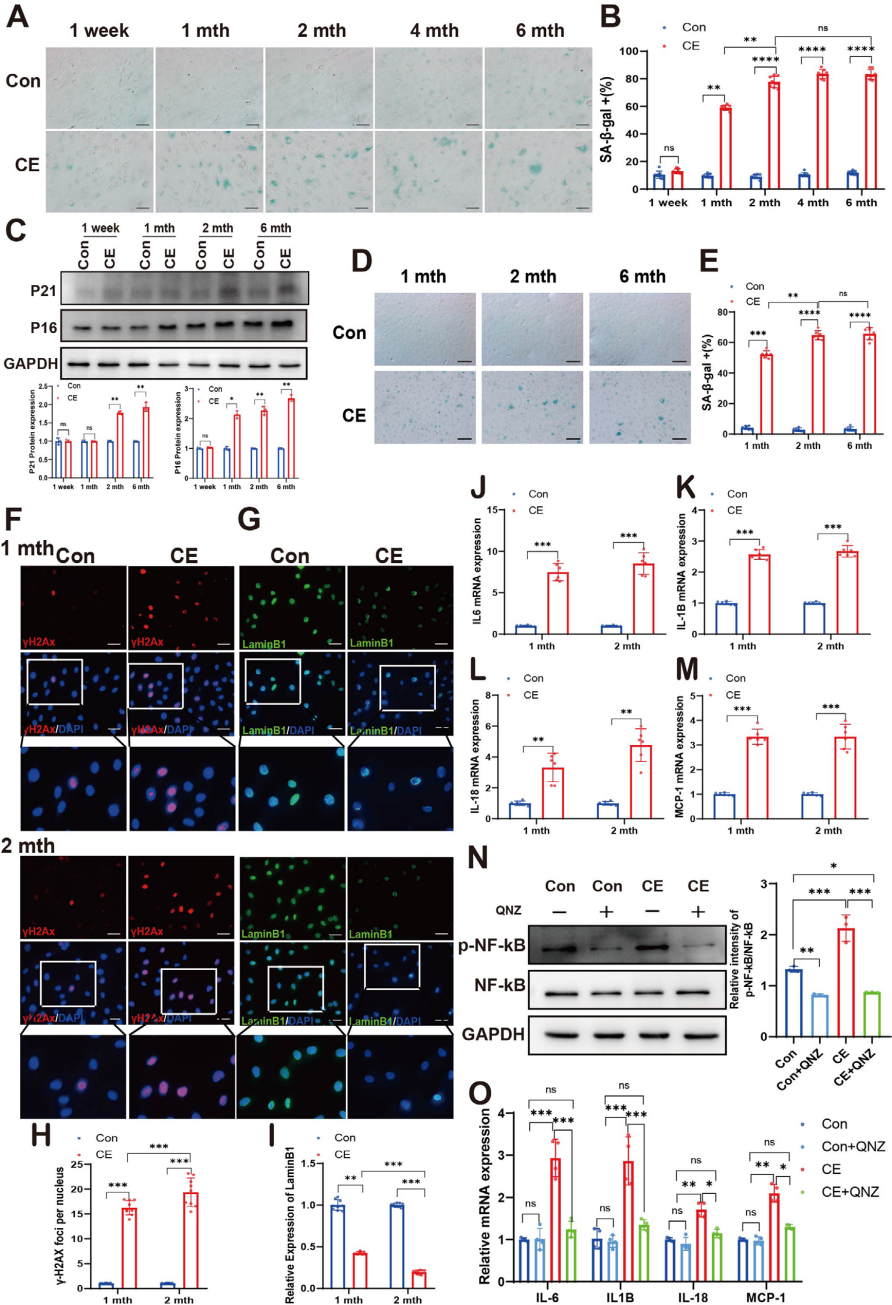
Plasma from cigarette exposure rats causes RPE to display a senescence phenotype. (A) SA‐β‐Gal staining in ARPE‐19 cells treated with plasma from normal air‐exposed rats (Con) and cigarette‐exposed rats (CE). Scale bar is 20 μm. (B) Quantitative analysis of SA‐β‐Gal positive cells in (A). (C) Western blot analysis and quantification of P21 and P16 expression levels in ARPE‐19 cells treated with plasma from normal air‐exposed rats (Con) and cigarette‐exposed rats (CE). (D) SA‐β‐Gal staining in hiPS‐RPE cells treated with plasma from normal air‐exposed rats (Con) and cigarette‐exposed rats (CE). Scale bar is 20 μm. (E) Quantitative analysis of SA‐β‐Gal positive cells in (D). (F) Representative images of γH2Ax in ARPE‐19 cells treated with plasma from the normal group of air‐exposed rats (Con) and cigarette‐exposed rats (CE), γH2Ax (red), and nuclear DNA stained with DAPI (blue). Scale bar is 20 μm. (G) Representative images of LaminB1 in ARPE‐19 cells treated with plasma from the normal group of air‐exposed rats (Con) and cigarette‐exposed rats (CE), LaminB1 (green), and nuclear DNA stained with DAPI (blue). Scale bar is 20 μm. (H) Quantitative analysis of fluorescence expression levels of γH2Ax in (F). (I) Quantitative analysis of fluorescence expression levels of LaminB1 in (G). (J) The expression of IL‐6, IL1B (K), IL18 (L) and MCP‐1 (M) in ARPE‐19 cells treated with plasma from the normal group of air‐exposed rats (Con) or cigarette‐exposed rats (CE) detected with qPCR. (N) Western blot analysis of NF‐kB and p‐NF‐kB expression treated with plasma from the normal group of air‐exposed rats (Con) or cigarette‐exposed rats (CE). (O) The effect of NF‐kB inhibitor QNZ on SASP (IL‐6, IL‐1B, IL‐18, MCP‐1) mRNA expression of plasma after cigarette exposure in ARPE‐19 cells was determined with qPCR. To ensure experimental robustness, three distinct plasma batches (*n* = 15 per batch) were independently processed and analyzed in all experimental procedures. *p* value measured by one‐way ANOVA and post hoc Bonferroni's test. Data are presented as means ± SD. Ns, not significant, *p < 0.05; **p < 0.01; ***p < 0.001; ****p < 0.0001.

We further established an in vitro model by treating ARPE‐19 cells with plasma from cigarette smoke‐exposed rats, enabling investigation of cigarette exposure effects at the cellular level. As shown in Figure 2A, ARPE‐19 cells treated with rat plasma collected 1 week post‐cigarette exposure exhibited limited SA‐β‐Gal‐positive staining, suggesting that even short‐term smoke exposure induces early senescence‐promoting effects at the cellular level. In addition, treatment with plasma collected from cigarette smoke‐exposed rats at 1‐month significantly increased SA‐β‐gal‐positive cells in ARPE‐19 cultures to 60% ± 2%. The duration of cigarette exposure prolonged, the inductive effect of plasma on cell aging was remarkably enhanced. Furthermore, when treated with the plasma collected after 2 months of cigarette exposure, the proportion of positive cells even rose to 80% ± 3%, fully reflecting the trend that the degree of cell aging continuously intensified with the increase in cigarette exposure time. Notably, treatment with plasma collected after four and 6 months of cigarette exposure resulted in a proportion of SA‐β‐gal‐positive ARPE‐19 cells comparable to that observed with plasma from 2 months of exposure, with no significant increase observed (Figure 2B). Subsequently, the results of detecting the senescence markers P21 and P16 showed that the plasma from 1 month of cigarette exposure promoted the increase in P16 expression but had no impact on P21. As the exposure time extended, the plasma from 2 months of cigarette exposure began to upregulate the expressions of both P16 and P21 (Figure 2C). Moreover, hiPS‐RPE cells treated with plasma from both the normal and cigarette‐exposed groups at various time points exhibited senescence staining patterns consistent with those observed in ARPE‐19 cells (Figure 2D,E). Thus, we selected the rat plasma from 1 and 2 months of cigarette exposure to carry out subsequent experiments.

Then, we detected other key indicators of senescent cells, namely γ‐H2Ax, LaminB1, and SASP. The results indicated that compared with the normal group, cigarette exposure significantly increased the expression of γ‐H2Ax and decreased the expression of LaminB1, and the effect was more pronounced after 2 months of cigarette exposure than after 1 month (Figure 2F–I). ARPE‐19 cells treated with plasma from cigarette‐exposed rats showed significantly increased SASP expression (Figure 2H–K). Subsequently, by using the NF‐κB inhibitor QNZ, we found that the plasma after cigarette exposure increased the expression of SASP through the NF‐κB signaling pathway (Figure 2J–O). All these results suggest that the rat plasma after cigarette exposure can effectively induce the senescence of ARPE‐19 cells in vitro.

## 3 Accumulation of Naphthalene From Smoking in Plasma Induces RPE Senescence

**FIGURE 3:**
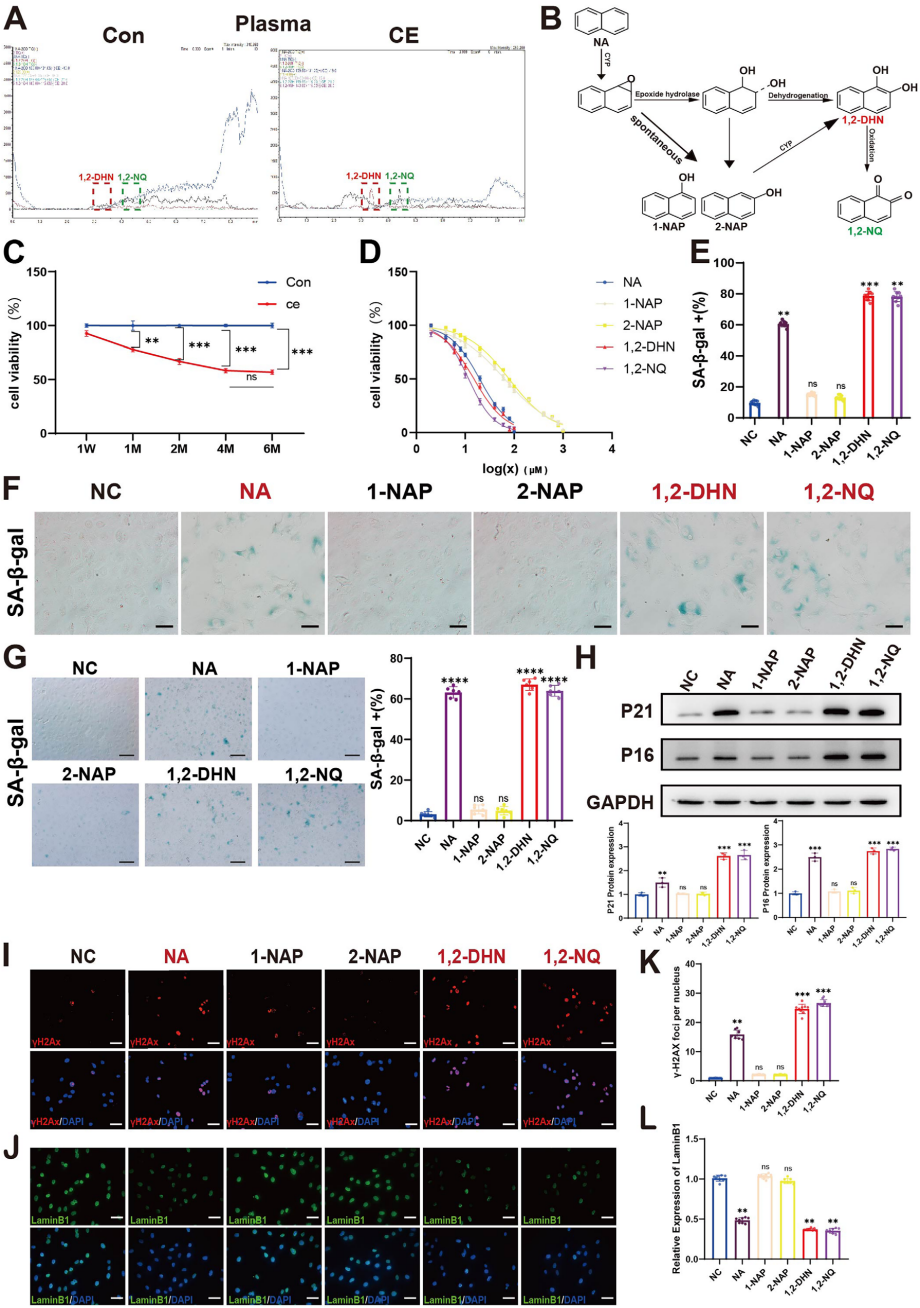
NA with its metabolites (1,2‐DHN and 1,2NQ) causes RPE to display a senescence phenotype. (A) Detect naphthalene metabolites in plasma from the normal group of air‐exposed rats (Con) and cigarette‐exposed rats (CE) by LC–MS. (B) Primary and secondary major metabolites of naphthalene. (C) Cell viability in ARPE‐19 cells after treatment with plasma from the normal group of air‐exposed rats (Con) or cigarette‐exposed rats (CE). To ensure experimental robustness, three distinct plasma batches (*n* = 15 per batch) were independently processed and analyzed in all experimental procedures. (D) Cell viability in ARPE‐19 cells after treatment with different concentrations of NA with its metabolites (1‐NAP, 2‐NAP, 1,2‐DHN, and 1,2‐NQ) by CCK8. *n* = 3. It can be seen that NA, 1, 2‐DHN, and 1, 2‐NQ are more toxic than 1‐NAP and 2‐NAP, and the toxicity of NA, 1, 2‐DHN, and 1, 2‐NQ increases successively. (E, F) SA‐β‐Gal staining and quantitative analysis of ARPE‐19 cells treated separately with NA and its metabolites (1‐NAP, 2‐NAP, 1,2‐DHN, and 1,2NQ). Scale bar is 20 μm. *n* = 3. (G) SA‐β‐Gal staining and quantitative analysis of hiPS‐RPE cells treated separately with NA and its metabolites (1‐NAP, 2‐NAP, 1,2‐DHN, and 1,2NQ). Scale bar is 20 μm. *n* = 3. (H) Western blot analysis of P21 and P16 expression levels in ARPE‐19 cells treated separately with NA and its metabolites (1‐NAP, 2‐NAP, 1,2‐DHN, and 1,2NQ). (I, J) Quantification of relative protein expression levels in (G). *n* = 3. (J) Representative images of γH2Ax and LaminB1 in ARPE‐19 cells treated separately with NA and its metabolites (1‐NAP, 2‐NAP, 1,2‐DHN and 1,2NQ), γH2Ax (red), LaminB1 (green), and nuclear DNA stained with DAPI (blue). Scale bar: 20 μm. (K) Quantitative analysis of fluorescence expression levels of γH2Ax in (J). *n* = 3. (L) Quantitative analysis of fluorescence expression levels of LaminB1 in (J). *n* = 3. Data are presented as means ± SD. *p* value measured by one‐way ANOVA and post hoc Bonferroni's test. Ns, not significant, *p < 0.05; **p < 0.01; ***p < 0.001; ****p < 0.0001.

Cigarette smoke exhibits an extremely complex chemical composition and harbors a multitude of toxic substances, among which are numerous polycyclic aromatic hydrocarbons (PAHs). The more abundant and lower molecular weight PAHs like naphthalene, fluorene, and phenanthrene predominantly contribute to the overall PAH yields, with naphthalene (NA) possessing the highest content among polycyclic aromatic hydrocarbons (Vu et al. 2015). Based on current research, we focused on naphthalene in cigarette smoke. Previous work showed that after cigarette exposure, 1,2‐dihydroxynaphthalene (1,2‐DHN) and 1,2‐naphthoquinone (1,2‐NQ), the metabolites of naphthalene, were detected in rat aqueous humor and retina. Now, we also detected 1,2‐DHN and 1,2‐NQ in rat plasma after cigarette exposure, while they were absent in the normal air exposure group (Figure 3A). Naphthalene, as a small‐molecule polycyclic aromatic hydrocarbon, can be metabolized in the body to form 1,2‐DHN, and then 1,2‐DHN can be further oxidized into 1,2‐NQ (Figure 3B). These two are typical toxic metabolites of naphthalene (Bogen et al. 2008; Wang, Bruyneel, et al. 2020). Next, we explored the roles of naphthalene and its metabolites in the process of ARPE‐19 cell senescence induced by cigarette exposure. Firstly, ARPE‐19 cells were treated with plasma from different durations of cigarette exposure for 24 h, and then cell viability was measured. Similarly, ARPE‐19 cells were treated with different concentrations of naphthalene and its metabolites for 24 h, and cell viability was detected by CCK‐8. Finally, according to the results in Figure 3C,D, When the concentration of 1,2‐NQ, the most toxic among naphthalene and its metabolites, was 10 μM, the degree of cell viability decline was similar to that of cells exposed to cigarette smoke for 2 months (both ~60%). Moreover, the detected doses of 1,2‐DHN and 1,2‐NQ in the plasma after cigarette smoke exposure were relatively close. Therefore, we selected 10 μM of naphthalene and its metabolites for subsequent experiments. At identical doses, naphthalene, 1,2‐DHN, and 1,2‐NQ increased senescent ARPE‐19 cell numbers, whereas 1‐NAP and 2‐NAP showed no effect (Figure 3E,F). Similarly, when hiPS‐RPE cells were treated with naphthalene and its metabolites, the senescence staining results were consistent with those observed in ARPE‐19 cells (Figure 3G). Moreover, in RPE cells, 10 μM naphthalene metabolites 1,2‐DHN and 1,2‐NQ induced senescent cell numbers comparable to those from long‐term cigarette‐exposed plasma.

The results in Figure 3H showed that the levels of P21 and P16 in cells treated with the metabolites of NA (1‐NAP and 2‐NAP) were consistent with those in the normal group, while NA and its metabolites (1,2‐DHN and 1,2‐NQ) significantly increased the protein expressions of P21 and P16. Compared with the normal group, the metabolites of NA (1‐NAP and 2‐NAP) had no effect on the expressions of γ‐H2Ax and LaminB1, whereas NA, 1,2‐DHN, and 1,2‐NQ increased the expression of γ‐H2Ax and decreased the expression of LaminB1 in ARPE‐19 cells (Figure 3I–L). Meanwhile, NA and its metabolites (1,2‐DHN and 1,2‐NQ) increased the secretion of SASP (IL‐6, IL‐1B, IL‐18, MCP‐1) in ARPE‐19 cells through the NF‐κB signaling pathway (Figure S2A,B). Subsequently, this result was further confirmed by using the NF‐κB inhibitor QNZ (Figure S2C,D). The above results indicate that, through the detection of cell senescence markers, the effects of NA, 1,2‐DHN, and 1,2‐NQ were highly consistent with those of plasma after cigarette exposure. In summary, through the systematic detection and analysis of multiple cellular senescence markers, we have found that NA, 1,2‐DHN, and 1,2‐NQ can induce a senescent phenotype in ARPE‐19 cells.

## 4 Cigarette Exposure Enhanced G2/M Cell Cycle Arrest and Promoted the Sustained Survival of Senescent Cells

Cellular senescence refers to the process in which previously replicative‐competent cells exit the cell cycle due to stress, subsequently leading to permanent cell‐cycle arrest (Zhang et al. 2022). During this process, the accumulation of senescent cells promotes the secretion of pro‐inflammatory cytokines, ultimately resulting in an increase in aging‐related diseases and morbidity. Notably, senescent cells can avoid apoptosis by means of cell‐cycle arrest and thus continue to survive (Hernandez‐Segura et al. 2018; Kuehnemann and Wiley 2024).

**FIGURE 4:**
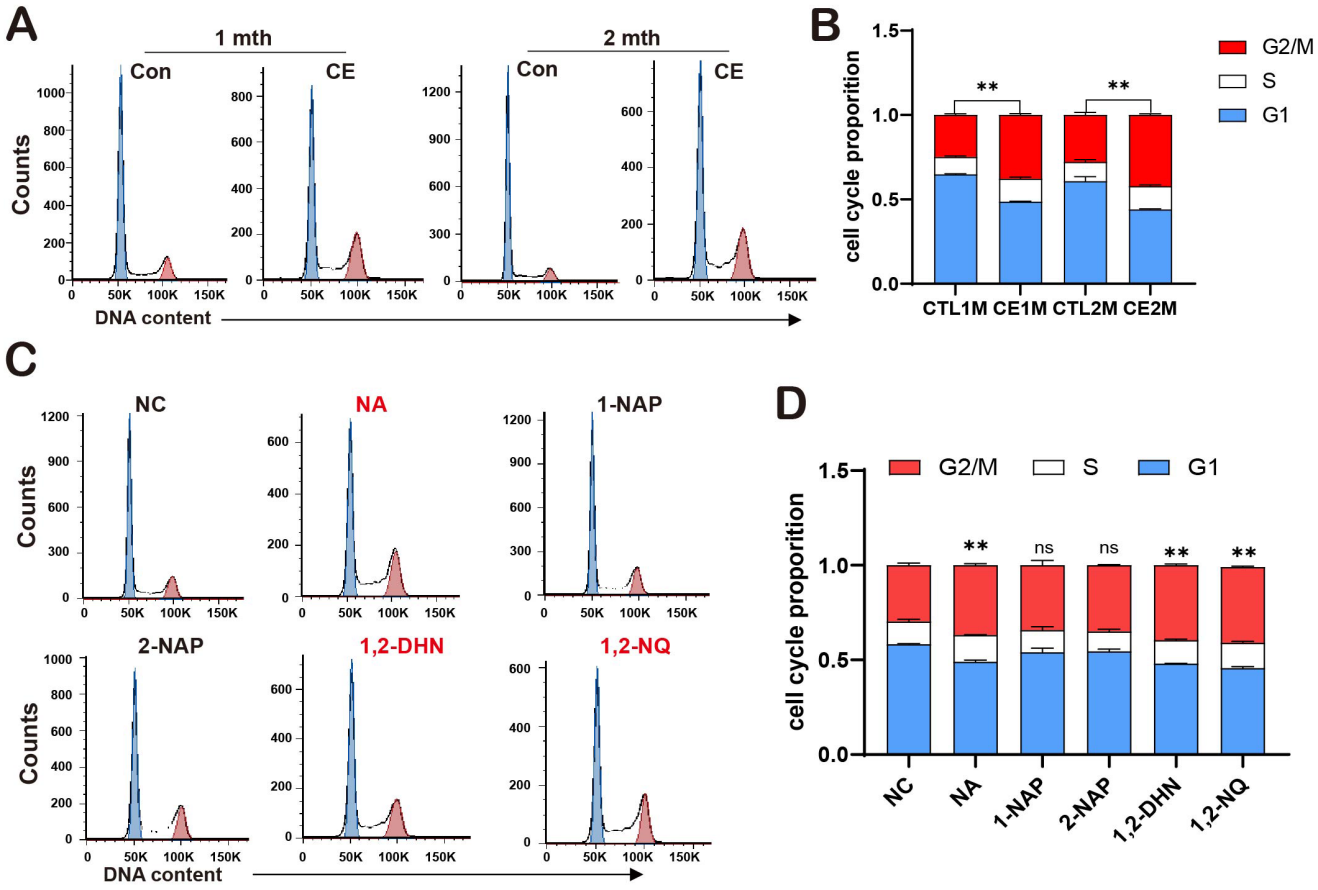
Plasma from cigarette‐exposed rats, NA and its metabolites (1,2‐DHN and 1,2‐NQ) lead to the arrest of ARPE‐19 cells in the G2/M phase. (A, B) Cell cycle analysis by propidium iodide staining and flow cytometry in ARPE‐19 cells treated with plasma from a normal group of air‐exposed rats (Con) and cigarette‐exposed rats (CE) for 24 h. To ensure experimental robustness, three distinct plasma batches (*n* = 15 per batch) were independently processed and analyzed in all experimental procedures. (C, D) Cell cycle analysis by propidium iodide staining and flow cytometry in ARPE‐19 cells treated separately with NA and its metabolites (1‐NAP, 2‐NAP, 1,2‐DHN, and 1,2‐NQ) for 24 h. *n* = 3. Data are presented as means ± SD. *p* value measured by one‐way ANOVA and post hoc Bonferroni's test. Ns, not significant; **p < 0.01.

In the study of the cigarette—exposure model, flow cytometry analysis revealed that after treating ARPE‐19 cells with the plasma of rats exposed to cigarettes for 1 or 2 months, the cells exhibited a G2/M—phase arrest phenomenon (Figure 4A,B). Further examination indicated that treatment with NA and its metabolites (1,2‐DHN and 1,2‐NQ) also caused ARPE‐19 cells to arrest at the G2/M phase. However, 1‐NAP and 2‐NAP had no impact on the cell cycle (Figure 4C,D).

In conclusion, both cigarette exposure and NA and its metabolites enhance the cell‐cycle arrest at the G2/M phase. This effect facilitates the continuous survival of senescent cells, enabling them to persist in the tissue microenvironment, which may potentially influence tissue homeostasis and overall physiological functions.

## 5 Long‐Term Cigarette Exposure Promotes RPE Cell Senescence by the P53 Signaling Pathway

**FIGURE 5:**
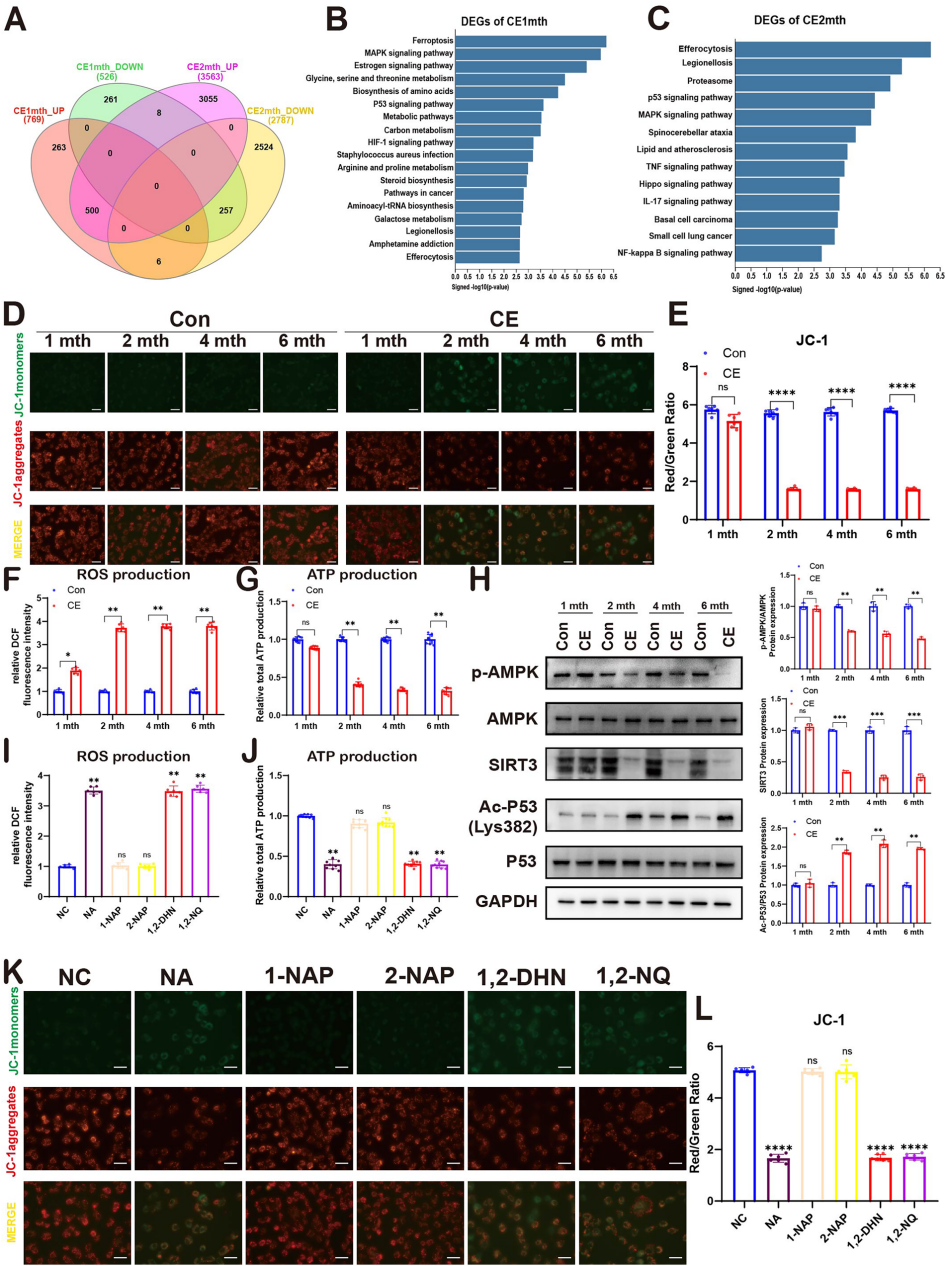
Long‐term cigarette smoke exposure induces mitochondrial dysfunction in the RPE of rats through plasma and NA as well as its metabolites (1,2‐DHN and 1,2‐NQ). (A) Venn diagram of DEGs of Con1 mtn, ce1 mth, Con2 mth, and ce2 mth. (B) Comparable analysis between Con1 mtn and ce1 mtn groups using the KEGG database. (C) Comparable analysis between Con2 mtn and ce2 mtn group using the KEGG database. (D, E) Immunofluorescence and quantification for mitochondrial membrane potential in ARPE‐19 cells treated with plasma from the normal group of air‐exposed rats (Con) and cigarette‐exposed rats (CE). Scale bar: 20 μm. (F) Fluorescence for ROS production in ARPE‐19 cells treated with plasma from the normal group of air‐exposed rats (Con) and cigarette‐exposed rats (CE). (G) ATP production in ARPE‐19 cells treated with plasma from the normal group of air‐exposed rats (Con) and cigarette‐exposed rats (CE). (H) Western blot analysis of AMPK, p‐AMPK, SIRT3, Ac‐P53 (Lys382) and P53 expression in ARPE‐19 cells treated with plasma from a normal group of air‐exposed rats (Con) and cigarette‐exposed rats (CE). (A–H) To ensure experimental robustness, three distinct plasma batches (*n* = 15 per batch) were independently processed and analyzed in all experimental procedures. (I) Fluorescence for ROS production in ARPE‐19 cells treated separately with NA and its metabolites (1‐NAP, 2‐NAP, 1,2‐DHN, and 1,2NQ). *n* = 3. (J) ATP production in ARPE‐19 cells treated separately with NA and its metabolites (1‐NAP, 2‐NAP, 1,2‐DHN, and 1,2NQ). *n* = 3. (K, L) Immunofluorescence and quantification for mitochondrial membrane potential in ARPE‐19 cells treated separately with NA and its metabolites (1‐NAP, 2‐NAP, 1,2‐DHN, and 1,2‐NQ). Scale bar: 20 μm; *n* = 3. Data are presented as means ± SD. *p* value measured by one‐way ANOVA and post hoc Bonferroni's test. Ns, not significant, *p < 0.05; **p < 0.01; ***p < 0.001; ****p < 0.0001.

After clarifying that cigarette smoke exposure and the metabolites of naphthalene (1,2‐DHN and 1,2‐NQ) can induce the aging of RPE, we applied RNA sequencing technology and exposed ARPE‐19 cells to the plasma of rats that had been exposed to cigarette smoke for 1 month (the early stage) or 2 months (the late stage), aiming to further explore the aging mechanism. Consistently, totally 1295 different expressed genes (DEG) (769 upregulated genes; 526 downregulated genes) were detected in the ARPE‐19 with plasma from 1 month. 6350 DEGs (3563 upregulated genes; 2787 downregulated genes) were detected for the plasma 2 months (Figure 5A). KEGG pathway analysis of DEGs of the 1‐month plasma‐treated ARPE‐19 cells shows ferroptosis, MAPK signaling pathway, estrogen signaling pathway, metabolism of amino acids, p53 signaling pathway, metabolic pathways, and HIF‐1 signaling pathway were involved in the regulation of RPE with early‐stage plasma. And the DEGs of the 2‐month plasma‐treated ARPE‐19 show proteasome, p53 signaling pathway, MAPK signaling pathway, lipid and atherosclerosis, TNF signaling pathway, IL‐17 signaling pathway, and NF‐kappa B signaling pathway (Figure 5B,C). Based on the above research findings, we first consider that ROS and mitochondrial dysfunction may play a crucial role in this system, accelerating the process of cellular senescence. This is because among the numerous signaling pathways involved in regulation that have been discovered, many are closely related to the generation and scavenging of ROS as well as the maintenance of mitochondrial function.

Next, we assessed the mitochondrial function of ARPE‐19 cells treated with plasma of cigarette exposure, NA, and NA's metabolites. ROS production of ARPE‐19 cells treated with plasma from rats with cigarette exposure for 1 month increased by about 2‐fold compared to the control group, but the MMP and ATP production were shown to have no significant change. With the plasma treated with cigarette exposure for 2 months or later, the ROS generation was significantly increased compared to the cells with plasma for 1 month, and both MMP and ATP were significantly decreased (Figure 5D–G). We consider the ROS production to be the first cellular metabolism of the ARPE‐19 cell response to the plasma with cigarette exposure. ROS plays a role in p53‐mediated cell senescence, and SIRT3 is a well‐known ROS inhibitor (Zhu et al. 2024). Meanwhile, AMPK can regulate the expression of SIRT3 and thus affect mitochondrial function (He et al. 2021; Wang et al. 2023; Yang, Wang, et al. 2022). All the expression of SIRT3, AMPK, and P53 in ARPE‐19 cells with 1 of month cigarette exposure plasma showed no significant difference. For the 2 months, AMPK and SIRT3 expression were significantly decreased, and levels of acetylated P53 protein increased in ARPE‐19 cells (Figure 5H). Similarly, after examining the RPE of rats treated with cigarette exposure, it was found that, in vivo, cigarette exposure can also significantly inhibit the activity of the AMPK/SIRT3 signaling pathway in rat RPE cells (Figure S3).

We also confirmed that NA, 1, 2‐DHN, and 1, 2‐NQ increased the production of ROS, decreased the production of ATP and MMP, and decreased the activity of AMPK and expression of SIRT3, and activated the acetylated P53 protein, while 1‐NAP and 2‐NAP had no effect on these proteins (Figure 5I–L; Figure S4). Collectively, these results clearly indicate that, compared with short‐term cigarette exposure, long‐term cigarette exposure not only triggers the initial metabolic change of intracellular ROS production, but also further leads to mitochondrial dysfunction by affecting the AMPK‐SIRT3 pathway. The impairment of mitochondrial function can give rise to a series of problems such as insufficient cellular energy supply and ROS accumulation, thus synergistically exacerbating the process of cellular senescence. Meanwhile, the mechanisms by which NA and its metabolites (1,2‐DHN and 1,2‐NQ) induce senescence in ARPE‐19 cells are highly consistent with those by which the plasma of cigarette‐exposed rats induces cellular senescence.

## 6 Stop Cigarette Exposure Ameliorating RPE Aging

Cigarette exposure surely leads to the RPE senescence by increasing the levels of 1,2‐DHN and 1,2‐NQ. We have to wonder if stop cigarette exposure can decrease the cellular toxicity and improve cell damage caused by short‐ or long‐term cigarette exposure, and the time cost of this accumulation of 1,2‐DHN and 1,2‐NQ will be eliminated. Sun et al. demonstrate that individuals with a long history of smoking can achieve a comparable health status to short‐term smokers by extending their stop cigarette exposure period (Cho et al. 2024; Sun et al. 2022).

**FIGURE 6:**
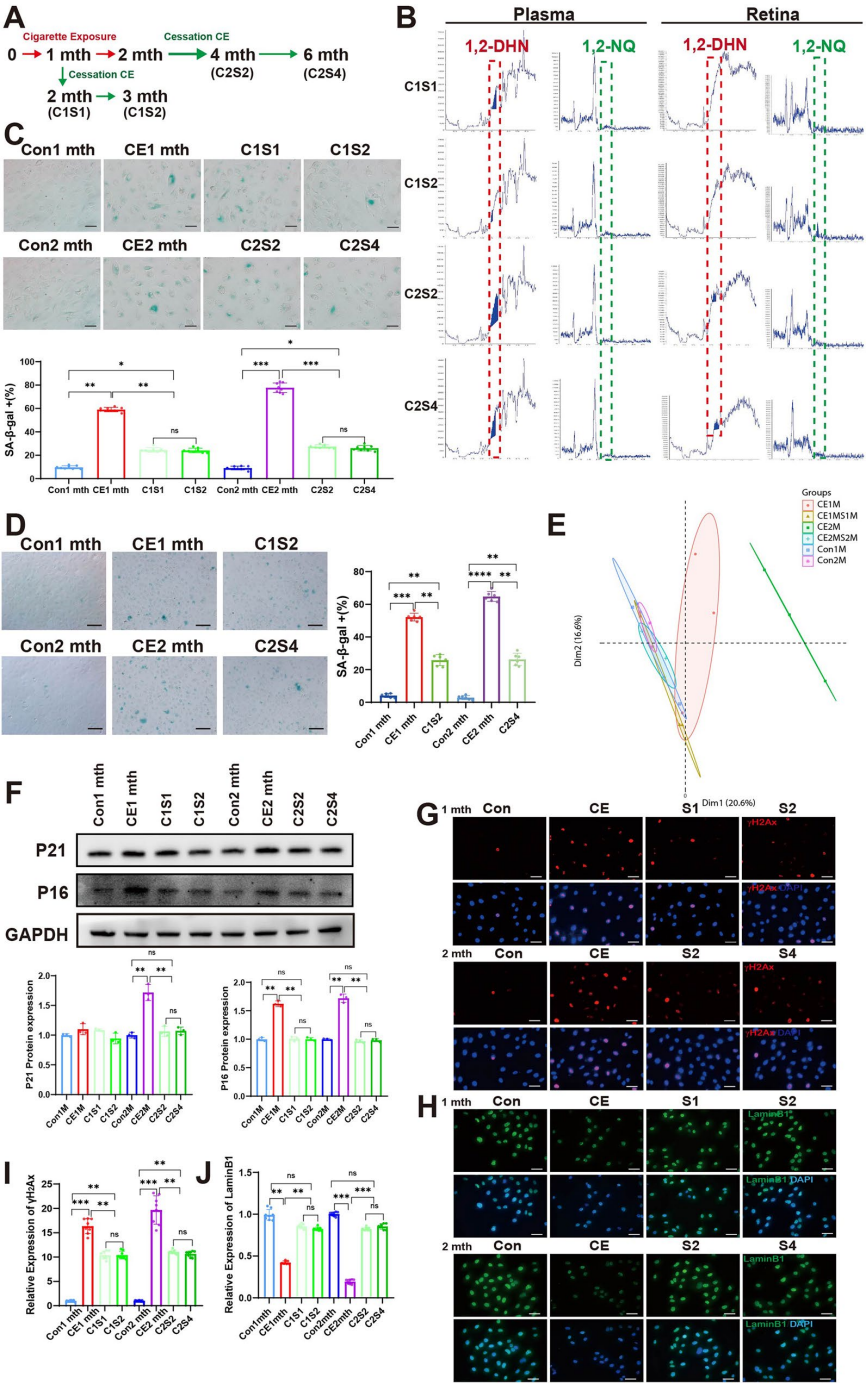
Smoking cessation can improve cell senescence caused by plasma exposed to cigarettes. (A) Outline of the smoking cessation model. (B) Detect 1,2‐DHN and 1,2‐NQ in plasma and retina from cessation smoking rats (CS) by LC–MS. (C) SA‐β‐Gal Staining in ARPE‐19 cells after smoking cessation. Scale bar: 20 μm. (D) SA‐β‐Gal Staining in hiPS‐RPE cells after smoking cessation. Scale bar: 20 μm. (E) A PCA plot shows the clusters of ARPE‐19 cells treated with plasma in different groups based on their similarity from RNA‐seq. (F) Western blot analysis and statistics of P21 and P16 expression in ARPE‐19 cells. (G–J) Representative images and statistics of γH2Ax and LaminB1 in ARPE‐19 cells, γH2Ax (red), LaminB1 (green), and nuclear DNA stained with DAPI (blue). Scale bar: 20 μm. To ensure experimental robustness, three distinct plasma batches (*n* = 15 per batch) were independently processed and analyzed in all experimental procedures. Data are presented as means ± SD. *p* value measured by one‐way ANOVA and post hoc Bonferroni's test. ns: Not significant, *p < 0.05; **p < 0.01; ***p < 0.001; ****p < 0.0001.

For this point, the rats were exposed to cigarettes for 1 month and then cessation for another 1 month (C1S1) or 2 months (C2S2) to study the improvement of stop cigarette exposure after short‐term cigarette exposure. And the rats were exposed to cigarettes for 2 months and then cessation for another 2 (C2S2) or 4 (C2S4) months to study the improvement of stop cigarette exposure after long‐term cigarette exposure (Figure 6A). The peak aeras of the 1,2‐DHN and 1,2‐NQ in plasma, aqueous humor, and retina were first detected by HPLC to evaluate the accumulation of these toxic chemicals from the cigarette exposure. As shown in Figure 6B; Figure S5, the C1S1 group was lower than the C2S2 groups, and the shorter cessation (C1S1 and C2S2) higher than the corresponding long‐term cessation (C1S2 and C2S4), respectively. The long‐term cigarette exposure will accumulate more 1,2‐DHN and 1,2‐NQ in the ocular circulation than the short‐term cigarette exposure and need more time to metabolize these toxic chemicals.

We further evaluate the toxicity of the plasma from these rats. Consistently, the percentage of SA‐β‐Gal positivity caused by short‐term cigarette exposure decreased significantly by ~40% after quitting smoking (*p* = 0.003). And during long‐term cigarette exposure, smoking cessation also significantly reduced the percentage of SA‐β‐Gal positivity by ~55% (*p* = 0.0002) (Figure 6C). Similarly, hiPS‐RPE cells treated with plasma from rats after cigarette cessation exhibited reduced SA‐β‐Gal positivie cells compared to those treated with plasma from cigarette‐exposed rats (Figure 6D). And ARPE‐19 cells treated with plasma after cigarette cessation are close to the state of cells with normal plasma based on the RNA sequencing (Figure 6E). Meanwhile, the increased expression of P21 and P16 also returns to normal levels (Figure 6F). In addition, both short‐term and long‐term stop cigarette exposure decreased irreparable DNA damage after quitting compared to the smoking group but was still higher than in the normal group. However, for both short‐term and long‐term cigarette exposure, the expression of LaminB1 after stop cigarette exposurewas no different from that in the normal group (Figure 6G–J). Moreover, plasma after stop cigarette exposure did not activate the NF‐kB pathway to increase SASP secretion, this suggests that the inflammatory response is greatly reduced after quitting smoking (Figure S6A–C).

**FIGURE 7:**
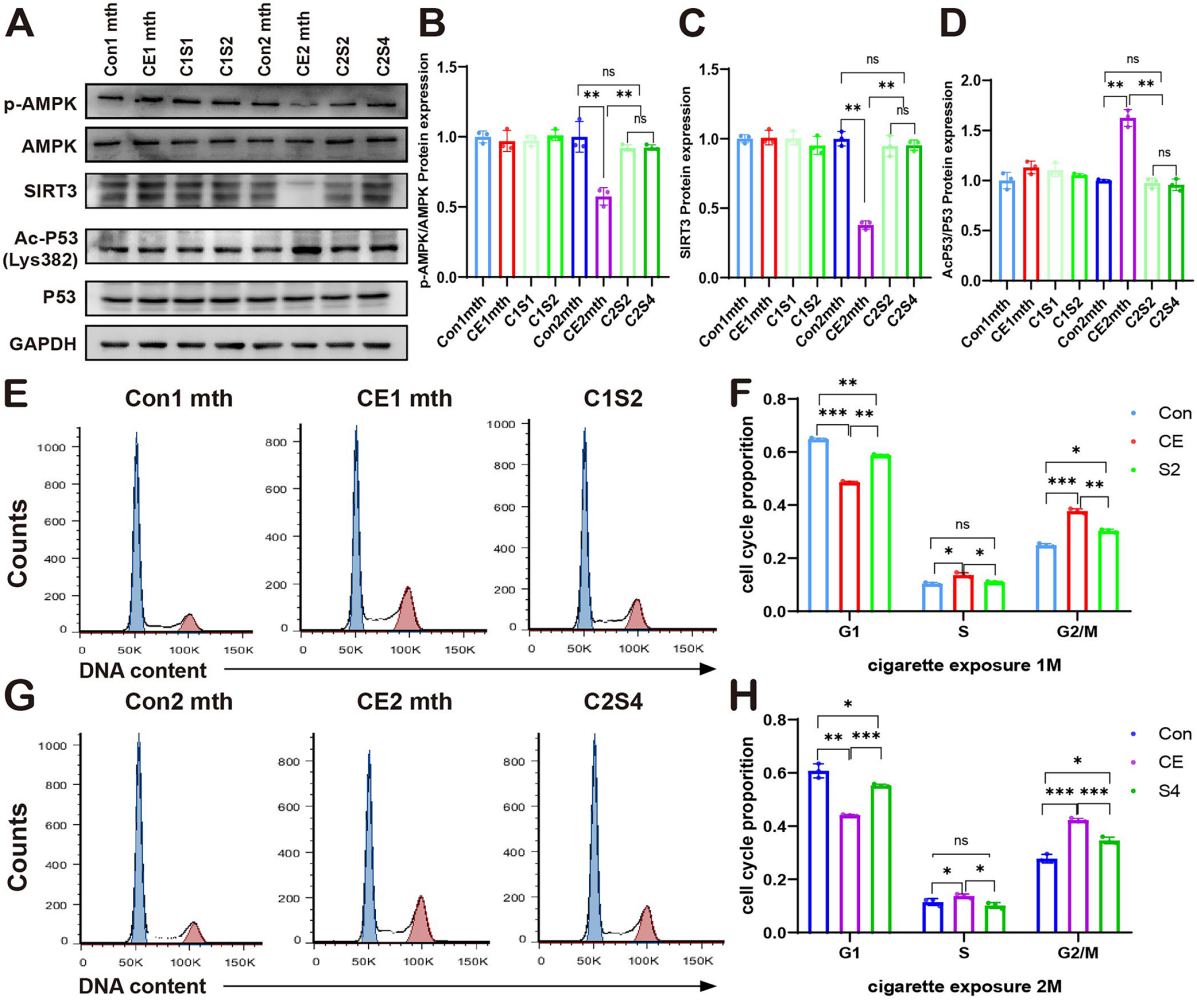
Smoking cessation can improve mitochondrial dysfunction and cell cycle arrest caused by plasma exposure to cigarettes. (A) Western blot analysis of SIRT3, Ac‐P53 (Lys382) and P53 expression in ARPE‐19 cells. (B–D) Quantification of relative protein expression levels in (A). (E, F) Cell cycle analysis by propidium iodide staining and flow cytometry in ARPE‐19 cells treated with plasma after short‐term exposure. (G, H) Cell cycle analysis by propidium iodide staining and flow cytometry in ARPE‐19 cells treated with plasma after long‐term exposure. To ensure experimental robustness, three distinct plasma batches (*n* = 15 per batch) were independently processed and analyzed in all experimental procedures. Data are presented as means ± SD. *p* value measured by one‐way ANOVA and post hoc Bonferroni's test. ns: Not significant, *p < 0.05; **p < 0.01; ***p < 0.001; ****p < 0.0001.

Importantly, plasma after cigarette exposure induced mitochondrial dysfunction due to decreased AMPK activity and SIRT3 expression, which were restored after stop cigarette exposure (Figure 7A–D; Figure S7A–D). Regardless of short‐term or long‐term exposure, stop cigarette exposure did not return the ARPE‐19 cell cycle to normal‐like status but improved cell arrest in the G2/M phase to some extent relative to cigarette exposure (Figure 7E–H).

In conclusion, stop cigarette exposure can improve the accumulation of naphthalene metabolites (1,2‐DHN and 1,2‐NQ) caused by cigarette exposure and the senescence of RPE. The earlier smoking cessation, the more conducive it will be to reducing the accumulation of toxic substances caused by smoking. However, regardless of whether it is after short‐term or long‐term cigarette exposure, there is little difference between long and short durations of smoking cessation.

## 7 Discussion

Tobacco use poses a significant public health problem and is the primary cause of deaths that could have been prevented globally (Galiatsatos et al. 2023). The harm of tobacco use mainly lies in the harmful substances contained in the smoke from burning cigarettes, including more than 70 carcinogens that can cause DNA damage (Tang et al. 2022). In recent years, a growing body of research has shown that DNA damage contributes to many aging phenotypes and may serve as a central factor driving the aging process (Schumacher et al. 2021). Little is known about the cellular mechanisms through which smoking contributes to the pathology of AMD.

Our study revealed that cigarette exposure significantly impairs retinal function in rats. γH2Ax staining demonstrated increased DNA damage in the ONL and RPE of rat retinas following cigarette exposure. After 4 months of exposure, RPE exhibited a senescence phenotype. While previous studies have identified histone loss as a unique feature of RPE senescence (Dubey et al. 2024), our cigarette exposure model showed that although total H2Ax protein levels remained unchanged, its phosphorylation level (i.e., γH2Ax) was significantly increased, indicating accumulation of unrepaired DNA damage. SASP in senescent RPE, characterized by increased secretion of immune regulators and proinflammatory factors, promotes the progression of AMD (Dubey et al. 2025). Consistently, this study found that cigarette exposure induced enhanced SASP secretion in RPE. Mechanistic investigations further revealed that cigarette exposure induced mitochondrial dysfunction by inhibiting the AMPK/SIRT3 signaling axis, ultimately driving RPE to exhibit classic senescence phenotypes. These findings provide critical experimental evidence for elucidating the cellular mechanisms underlying smoking‐related AMD.

The acceleration of aging caused by smoking may be attributed to the fact that the cigarette combustion process generates a wide variety of harmful substances with complex compositions. The main harmful substances in cigarette smoke are PAHs, among which naphthalene has the highest content (Vu et al. 2015). Moreover, cigarette smoke has a significant influence on human exposure to naphthalene. There is a highly significant correlation between the level of 2‐naphthol in the urine of smokers and the number of cigarettes smoked per day (reflected by the level of urinary cotinine) (Preuss et al. 2003). Previous study has shown that naphthalene metabolites can induce cellular damage through multiple mechanisms (Cui et al. 2025). In this study, naphthalene and its metabolites (1,2‐DHN and 1,2‐NQ) induced senescence in ARPE‐19 cells‐a phenotype consistent with that caused by plasma from cigarette‐exposed rats. These findings suggest that controlling naphthalene levels in cigarette smoke may mitigate smoking‐related cellular damage. Zhao et al. has demonstrated that *Burkholderia cepacia* (BK) immobilized on reduced graphene oxide can remove 99.0% of naphthalene within 48 h (Zhao et al. 2023).

Smoking cessation may have a protective effect against the risk of age‐related macular degeneration (Kulkarni and Banait 2023). However, it is still difficult for smokers to make the decision to stick to quitting. In our study, it is worth emphasizing that whether cigarette exposure is short‐ or long‐term, as long as after a period of stop cigarette exposure, by detecting senescence markers, we found that RPE senescence due to cigarette exposure can be alleviated after smoking cessation. Our findings underscore the profound benefits of cessation smoking for retinal health. We would also like to develop devices that filter naphthalene metabolites if they are available, for example, filtering cigarette mouthpieces. In addition, recent studies have demonstrated that the concentration of chemicals in e‐cigarette vapor is significantly lower—by several orders of magnitude—compared to that found in traditional cigarette smoke (Warner et al. 2023). The use of e‐cigarettes, a substitute for traditional smoking, might help smokers quit completely and quickly, but they are not currently approved for smoking cessation. But e‐cigarettes are also risky for health, and current studies have linked e‐cigarette use to an increased risk of cardiovascular disease, chronic obstructive pulmonary disease, cancer, oral disease, etc. (Afolabi and Rao 2023; Huang et al. 2024; Sahu et al. 2023).

In the end, our research shows that cigarette exposure raises the levels of 1,2‐DHN and 1,2‐NQ and lowers the function of the retina by triggering RPE senescence. Therefore, reducing the production of naphthalene in the cigarette production process can reduce the harm of smoking. Moreover, stop cigarette exposure can mitigate the negative effects of smoking on the RPE. Although stop cigarette exposure cannot completely reverse the damage already caused by senescence, it can prevent further harm. Finally, we recommend implementing routine smoking cessation counseling for AMD patients in clinical practice. Meanwhile, great attention should also be paid to the hazards brought by ETS.

## 8 Materials and Methods

### 8.1 Ethics Approval

The animal research program was reviewed and authorized by the experimental animal ethics committee of Tongji University (TJAA09620208). The SD (Sprague–Dawley) rats were purchased from Shanghai SLAC Laboratory Animal Company (Shanghai, China). SD rats (8 weeks) were selected and adaptively fed.

### 8.2 Cigarette Exposure Animal Model

The shell of the smoke box and the air pipe are made of environment‐friendly resin. A rubber ring is used to seal around the cover of the box, and the airflow in the box is realized through the front and rear two one‐way air valves. As shown in Figure 1A, the cigarette continued to produce smoke after it was ignited in the gas chamber. The smoke box was also provided with a feed stand and a drinking stand for the rats to access freely. In addition, a “multi‐gas data acquisition and calculation system” (Yuanyi Electronic Technology Co. Ltd., Shanghai, China) was added into the smoke box. The data collected by the sensor can be analyzed by chip to reflect the temperature and humidity, oxygen, carbon monoxide, and PM2.5 content in the smoke chamber in real time, so as to guide the dose and practice of cigarette exposure.

The rats were randomly divided into three groups: normal control group, cigarette exposure group, and cigarette exposure cessation group. The control rats were fed in the normal environment. The rats in the cigarette exposure group were exposed to a 10/84 mm cigarette (tar: 11 mg; nicotine: 1 mg; carbon monoxide: 13 mg; Double Happiness, Shanghai Tobacco Corporation, China) for 6 h every day, 5 days a week. The rats in the smoking cessation group stopped exposure after cigarette exposure.

### 8.3 Tissue Collection of Experimental Animals

For the plasma extraction, after intraperitoneal injection of pentobarbital sodium anesthesia, the thorax of rats was opened, and the blood was collected in the tube where heparin was added in advance using the method of apical blood collection. Following centrifugation (1500 rpm, 30 min, 4°C), the supernatant was transferred to a fresh tube, flash‐frozen in liquid nitrogen to halt enzymatic activity, and stored at −80°C.

For the extraction of eye tissues, the eyes of rats were dissected with a sterilized ophthalmological soak and rinsed with PBS. Then the eyes were fixed in a 4% paraformaldehyde (PFA; E672002‐0500, Sangon Biotech, China) solution for 12 h and sucrose gradient dehydration. The eyes were embedded with Tissue‐Tek OCT compound and cut into 10‐μm‐thick cryosections.

### 8.4 Flash Electroretinogram (f‐ERG) Recording

ERG recording was performed using the APS‐2000 visual electrophysiological examination system (Kanghuaruiming S&T, Chongqing, China). All animals were dark‐adapted overnight (12 h) and then ERG examination was performed under dim red light at the flash intensity of 6.325 e^−2^ cd × s/m after placing corneal contact electrodes and midline sub‐dermal reference and ground electrodes.

### 8.5 SA‐β‐Gal Staining

The Senescence‐associated β‐gal Assay Kit (C0602, Beyotime, China) was used, and SA‐β‐gal staining was conducted according to the manufacturer's instructions. For ARPE‐19 cells seeded in culture plates, the medium was removed, and the cells were fixed with the provided fixing solution for 15 min at room temperature. Then, the cells were washed three times for 5 min each time with PBS and incubated with freshly prepared SA‐β‐gal staining solution at 37°C for 6 h without CO_2_ and in the dark. The frozen sections were fixed with fixing solution for 15 min and washed with PBS 3 times for 5 min each time. Then, the sections were stained with SA‐β‐gal staining solution in an incubator at 37°C for 12 h without CO_2_ and in the dark. Senescent cells were observed under a light microscope (Olympus, Tokyo, Japan) and evaluated by counting the number through staining the nuclei with diamino‐2‐phenylindoline dihydrochloride.

### 8.6 Immunofluorescence

The retinal sections and cells were immobilized, soaked with 0.1% Triton X‐100 (A417820‐0100, Sangon Biotech, China) for 5 min, cleaned with PBS, and then sealed with 3% bovine serum albumin (BSA; A600332, Sangon Biotech, China) in PBS. The sections were incubated with the primary antibodies (γH2Ax and LaminB1) at 4°C overnight and then incubated with Alexa Fluor 488/555‐conjugated secondary antibodies (Table S1) for 1 h at room temperature in the dark. The nucleus was labeled with 4′,6‐diamidino‐2‐phenylindole (DAPI; D9542, Sigma, Germany). The sections were then examined by a fluorescence microscope (DP73, Olympus, Tokyo, Japan).

### 8.7 Liquid Chromatography‐Mass Spectrometry (LC–MS)

For the collection of retina and aqueous humor, the eyeballs were dissected and rinsed with sterilized ddH_2_O. The eyeballs were drained with autoclaved filter paper and placed in a new petri dish. The aqueous solution was collected by the “anterior chamber puncture method” under the stereoscopic microscope. The retina and RBCC were removed sequentially. And then put into liquid nitrogen for quick‐freezing, and stored at −80°C.

For the experimental pretreatment of samples for LC–MS, 100 μL of mobile phase with 0.2% glacial acetic acid (volume fraction) for every 10 mg of solid tissue (retina). For every 10 μL of liquid samples (plasma, aqueous humor), 20 μL of glacial acetic acid are added in advance by 0.2% (volume fraction) of the mobile phase. The tissue homogenization was recorded at 4°C and 65 Hz in 2 min intervals after dilution. During this period, the homogenizer is lightly elastic so that the substance to be measured can be fully extracted by the mobile phase. After centrifugation at 4°C at 12000 rpm for 15 min, the supernatant after centrifugation was taken and loaded into a new centrifuge tube. Dilution was performed by adding mobile phase at a ratio of 1:2 to further reduce matrix effect, and dilution multiples were recorded. Finally, acetamide was added, the final concentration was 0.4, and the sample was rapidly injected for analysis.

### 8.8 Cell Culture

ARPE‐19 cells were cultured in Dulbecco's Modified Eagle Medium: Nutrient Mixture F‐12 (DMEM/F‐12, D8437, Sigma, Germany) supplemented with 10% fetal bovine serum (F0193, Sigma, Germany) at 37°C, 5% CO_2_. Then the ARPE‐19 cells were passaged to several groups, which were treated with the plasma of rats for the next analysis.

hiPS‐RPE cells, originally derived as described by (Zhu et al. 2022), were dissociated using 0.25% trypsin/0.53 mM EDTA and then cultured with DMEM/F‐12 medium supplemented with 10% fetal bovine serum on dishes precoated with 1% Matrigel (diluted in DMEM/F12 medium; #354234, Corning, USA).

### 8.9 RNA Sequencing

The total RNA of cultured ARPE‐19 cells was isolated with TRIzol reagent (9109, Takara, Japan). The library was sequenced with an Illumina NovaSeq 6000 PE150. The criteria |log_2_FC| > 1 and *p* < 0.05 were applied to filter the differentially expressed genes. Gene ontology (GO) and Kyoto Encyclopedia of Genes and Genomes (KEGG) analyses were performed and visualized with WebGestalt (Elizarraras et al. 2024). RNA‐seq data generated in the study can be accessed at the Gene Expression Omnibus under accession code GSE276821 (https://www.ncbi.nlm.nih.gov/geo/query/acc.cgi?acc=GSE276821).

### 8.10 Western Blot Analysis

The cells were collected with RIPA (C0045, TargetMol, USA) with the addition of protease and phosphatase inhibitors (C0001 and C0004, TargetMol, USA), and the protein concentration was determined. An equal amount of protein is dissolved on the SDS‐polyacrylamide gel and transferred to the polyvinylidene fluoride (PVDF; IPVH00010, Sigma, Germany) membrane. The membrane was sealed in PBS with 5% BSA at room temperature for 1 h and incubated with the primary antibodies (Table S1) at 4°C overnight. After that, the membranes were incubated with the corresponding secondary antibodies (Table S1) at room temperature for 1.5 h and then washed three times with TBST. Finally, treated with chemiluminescence imaging (Tanon5200, Shanghai, China). Quantification was performed with ImageJ software (Version 1.48v).

### 8.11 ROS Production

The Reactive Oxygen Species Assay Kit (S0033S, Beyotime, China) was used. The fluorescence probe DCFH‐DA was used to detect reactive oxygen species. DCFH‐DA itself has no fluorescence and can freely pass through the cell membrane. After entering the cell, DCFH can be hydrolyzed by the esterase in the cell. DCFH cannot permeate the cell membrane, making it easy for the probe to be loaded into the cell. Intracellular reactive oxygen species can oxidize non‐fluorescent DCFH to produce fluorescent DCF. Detecting the fluorescence of DCF can determine the level of intracellular reactive oxygen species. Finally, the excitation wavelength of 488 nm and the emission wavelength of 525 nm were used to detect the intensity of fluorescence.

### 8.12 ATP Production

Intracellular ATP (adenosine 5′‐triphosphate) levels were detected using an ATP Assay Kit (S0027, Beyotime, China). The operation was carried out according to the instructions. After BCA quantification, the protein concentration of all groups to be measured was consistent. Finally, chemiluminescence signals were quantified at a 0.2s exposure time using a chemiluminescence detection system for statistical analysis.

### 8.13 Mitochondrial Membrane Potential Detection

The levels of mitochondrial membrane potential (MMP) were detected using a JC‐1 Assay Kit (C2006, Beyotime, China). After the cells were treated with plasma or naphthalene and its metabolites, they were washed once with PBS. The JC‐I reagent was diluted to 10 μM with DMEM/F12 medium and then added to the cells and incubated in the incubator for 20 min. Remove JC‐1 and wash once with PBS. After adding the medium, the red fluorescence and green fluorescence were observed under a fluorescence microscope, and photos were taken. At the same time, the excitation light can be set to 490 nm and the emission light can be set to 530 nm when the fluorescence enzyme is used to detect JC‐1 monomer. When detecting the JC‐1 polymer, the excitation light can be set to 525 nm and the emission light can be set to 590 nm.

### 8.14 Cell Viability Assay

Cell viability was measured by using the Cell Counting Kit‐8 assay (C0005, TargetMol, USA). The cells were seeded on 96‐well plates at a density of 1.0 × 10^4^ cells per well. After stimulation, the working solution was added and incubated in the incubator for 2 h and measured by recording the absorbance at 450 nm with a microplate reader (iMark Microplate Absorbance Reader, Bio‐Rad, USA).

### 8.15 Cell Cycle Detection

The cell cycle of ARPE‐19 cells was detected by flow cytometry using PI/RNase staining (C1052, Beyotime, China). ARPE‐19 cells were collected and fixed in 70% cold ethanol overnight at 4°C. Then, the ARPE‐19 cells were incubated with PI (5 μg/mL) and RNase A (1 mg/mL) in 500 μL of PBS at 37°C in the dark. Thirty minutes later, flow cytometry was performed, and the data were analyzed using FlowJo V10.

### 8.16 Quantitative Real‐Time Polymerase Chain Reaction (qRT‐PCR)

Total RNA was extracted, and reverse transcription was performed using the Primescript TM RT Master Mix kit (Takara, Shiga, Japan). qRT‐PCR was performed in a Chromo4 instrument cycler (CFX96, Bio‐Rad, Hercules, USA) using the Superreal Premix Plus kit (Tiangen Biotech, Beijing, China). PCR amplification was carried out with the following cycling parameters: denaturation at 95°C for 5 min, followed by 40 cycles of 95°C for 30 s and 60°C for 30 s. GAPDH served as the reference gene, and the relative gene expression was determined with the 2^−ΔΔCt^ method. The relevant primers are listed in Table S2.

### 8.17 Statistical Analyses

Data were analyzed using GraphPad Prism 10 software (GraphPad Software, USA). All data were expressed as means ± standard deviation. Differences between two groups were assessed with the two‐tailed Student's unpaired *t*‐test. One‐way analysis of variance (ANOVA) with the post hoc Bonferroni's test was used for multi‐group comparisons. Statistical significance was set at *p* < 0.05.

## Author Contributions

T.C., Q.O., C.J., J.‐Y.X., and G.‐T.X. conceived and designed the study and wrote the manuscript. J.X., Y.Z., and Y.L. collected samples. J.Z. and C.J. contributed to the materials. Z.W., Y.B., X.J., F.G., L.L., and J.W. made valuable suggestions regarding this study and co‐supervised the writing of the paper. Q.O., H.T., J.‐Y.X., and G.‐T.X. directed and supervised the study. All authors contributed to the manuscript and approved the submitted version.

## Conflicts of Interest

The authors declare no conflicts of interest.

## Supporting information


**Figure S1.** Diagram of oxygen and carbon dioxide concentrations in a cigarette exposure model box.
Change of O_2_ and CO_2_ concentration in self‐made smoker. Each collection point is 30 min apart.
**Figure S2.** NA with its metabolites (1,2‐DHN and 1,2‐NQ) increases SASP secretion through the NF‐kB signaling pathway. (A) The expression of IL‐6, IL‐1B, IL‐18, and MCP‐1 in ARPE‐19 cells treated separately with NA and its metabolites (1‐NAP, 2‐NAP, 1,2‐DHN, and 1,2NQ). (B) Western blot analysis of NF‐kB and p‐NF‐kB expression in ARPE‐19 cells treated separately with NA and its metabolites (1‐NAP, 2‐NAP, 1,2‐DHN and 1,2‐NQ). *n* = 3. (C) Western blot analysis of NF‐kB and p‐NF‐kB expression in ARPE‐19 cells after treatment with NF‐kB inhibitor QNZ. *n* = 3. (D) The effect of NF‐kB inhibitor QNZ on SASP (IL‐6, IL‐1B, IL‐18, and MCP‐1) mRNA expression in ARPE‐19 cells treated separately with NA and its metabolites (1‐NAP, 2‐NAP, 1,2‐DHN, and 1,2NQ). Data are presented as means ± SD. *p* value measured by one‐way ANOVA and post hoc Bonferroni’s test. Ns, not significant, NA, 1,2‐DHN, 1,2‐NQ, NC+QNZ, NA+QNZ, 1,2‐DHN+QNZ or 1,2‐NQ+QNZ versus NC *p < 0.05; **p < 0.01; ***p < 0.001; ****p < 0.0001. NA + QNZ versus NA ^####^ *p* < 0.0001, ^###^ *p* < 0.001, ^##^ *p* < 0.01, ^#^ *p* < 0.5. 1,2‐DHN + QNZ versus 1,2‐DHN ^$$$$^ *p* < 0.0001, ^$$$^ *p* < 0.001, ^$$^ *p* < 0.01, ^$^ *p* < 0.5. 1,2‐NQ + QNZ versus 1,2‐NQ ^++++^ *p* < 0.0001, ^+++^ *p* < 0.001, ^++^ *p* < 0.01, ^+^ *p* < 0.5.
**Figure S3.** The inhibitory effect of cigarette exposure on the activity of AMPK/SIRT3 in RPE of rats. Western blot was used to analyze the expressions of AMPK, p‐AMPK, SIRT3, Ac‐P53 (Lys382) and P53 in RBCC of normal group and cigarette exposure group. *n* = 3. Data are presented as means ± SD. *p* value measured by one‐way ANOVA and post hoc Bonferroni’s test. Ns, not significant, *p < 0.05; **p < 0.01; ***p < 0.001; ****p < 0.0001.
**Figure S4.** NA with its metabolites (1,2‐DHN and 1,2‐NQ) induces cellular senescence in ARPE‐19 cells through the P53 pathway. Western blot analysis of AMPK, p‐AMPK, SIRT3, Ac‐P53 (Lys382) and P53 expression in ARPE‐19 cells treated separately with NA and its metabolites (1‐NAP, 2‐NAP, 1,2‐DHN and 1,2NQ). *n* = 3. Data are presented as means ± SD. *p* value measured by one‐way ANOVA and post hoc Bonferroni’s test. Ns, not significant, *p < 0.05; **p < 0.01; ***p < 0.001; ****p < 0.0001.
**Figure S5.** Smoking cessation can reduce the content of 1,2‐DHN and 1,2‐NQ in the aqueous humor of rats. Detect 1,2‐DHN and 1,2‐NQ in plasma, aqueous humor, and retina from smoking cessation rats by LC–MS.
**Figure S6.** Smoking cessation can inhibit the activation of NF‐kB and thus reduce the secretion of inflammatory factors. (A) Western blot analysis and statistics of NF‐kB and p‐NF‐kB expression in ARPE‐19 cells. (B, C) The expression of SASP (IL‐6, IL‐1B, IL‐18, MCP‐1) mRNA levels was detected with qPCR. To ensure experimental robustness, three distinct plasma batches (*n* = 15 per batch) were independently processed and analyzed in all experimental procedures. Data are presented as means ± SD. *p* value measured by one‐way ANOVA and post hoc Bonferroni’s test. ns: not significant, *p < 0.05; **p < 0.01; ***p < 0.001; ****p < 0.0001.
**Figure S7.** Smoking cessation can improve mitochondrial dysfunction caused by cigarette exposure. (A, B) Immunofluorescence for mitochondrial membrane potential in ARPE‐19 cells treated with plasma from a normal group of air‐exposed rats (Con), cigarette‐exposed rats (CE) and cessation smoking (CS). Scale bar: 20 μm. (C) Fluorescence for ROS production in ARPE‐19 cells. (D) ATP production in ARPE‐19 cells. To ensure experimental robustness, three distinct plasma batches (*n* = 15 per batch) were independently processed and analyzed in all experimental procedures. Data are presented as means ± SD. *p* value measured by one‐way ANOVA and post hoc Bonferroni’s test. Ns, not significant, *p < 0.05; **p < 0.01; ***p < 0.001; ****p < 0.0001.

**Table S1.** Antibodies used in immunostaining and WB.

**Table S2.** Primers used in RT‐PCR experiments.

## Data Availability

All data shown in this work are available from the authors upon request.

## References

[acel70150-bib-0001] Abusharkh, F. H. , L. Kurdi , R. W. Shigdar , R. A. Mandura , and K. Alattas . 2023. “Prevalence and Associated Risk Factors of Age‐Related Macular Degeneration in the Retina Clinic at a Tertiary Center in Makkah Province, Saudi Arabia: A Retrospective Record Review.” Cureus 15, no. 3: e36048. 10.7759/cureus.36048. 37056542 PMC10089638

[acel70150-bib-0002] Afolabi, F. , and D. R. Rao . 2023. “E‐Cigarettes and Asthma in Adolescents.” Current Opinion in Allergy and Clinical Immunology 23, no. 2: 137–143. 10.1097/aci.0000000000000891. 36821483

[acel70150-bib-0003] Belmouhand, M. , S. P. Rothenbuehler , J. Bjerager , et al. 2022. “Heritability and Risk Factors of Incident Small and Large Drusen in the Copenhagen Twin Cohort Eye Study: A 20‐Year Follow‐Up.” Ophthalmologica 245, no. 5: 421–430. 10.1159/000525652. 35878587

[acel70150-bib-0004] Blasiak, J. , P. Sobczuk , E. Pawlowska , and K. Kaarniranta . 2022. “Interplay Between Aging and Other Factors of the Pathogenesis of Age‐Related Macular Degeneration.” Ageing Research Reviews 81: 101735. 10.1016/j.arr.2022.101735. 36113764

[acel70150-bib-0005] Bogen, K. T. , J. M. Benson , G. S. Yost , et al. 2008. “Naphthalene Metabolism in Relation to Target Tissue Anatomy, Physiology, Cytotoxicity and Tumorigenic Mechanism of Action.” Regulatory Toxicology and Pharmacology 51, no. 2 Suppl: S27–S36. 10.1016/j.yrtph.2007.10.018. PMC4030291 18191315

[acel70150-bib-0006] Cabral de Guimaraes, T. A. , M. Daich Varela , M. Georgiou , and M. Michaelides . 2022. “Treatments for Dry Age‐Related Macular Degeneration: Therapeutic Avenues, Clinical Trials and Future Directions.” British Journal of Ophthalmology 106, no. 3: 297–304. 10.1136/bjophthalmol-2020-318452. 33741584 PMC8867261

[acel70150-bib-0007] Cho, E. R. , I. K. Brill , I. T. Gram , P. E. Brown , and P. Jha . 2024. “Smoking Cessation and Short‐ and Longer‐Term Mortality.” NEJM Evidence 3, no. 3: EVIDoa2300272. 10.1056/EVIDoa2300272. 38329816

[acel70150-bib-0008] Cui, T. , Y. Liu , F. Gao , et al. 2025. “Asparagine Alleviates Naphthalene‐Induced Lens Opacity by Suppressing Ferroptosis.” Experimental Eye Research 255: 110362. 10.1016/j.exer.2025.110362. 40147683

[acel70150-bib-0009] Deng, Y. , L. Qiao , M. Du , et al. 2022. “Age‐Related Macular Degeneration: Epidemiology, Genetics, Pathophysiology, Diagnosis, and Targeted Therapy.” Genes Dis 9, no. 1: 62–79. 10.1016/j.gendis.2021.02.009. 35005108 PMC8720701

[acel70150-bib-0010] Dubey, S. K. , R. Dubey , K. Jung , A. G. Hernandez , and M. E. Kleinman . 2025. “Deciphering Age‐Related Transcriptomic Changes in the Mouse Retinal Pigment Epithelium.” Aging (Albany NY) 17, no. 3: 657–684. 10.18632/aging.206219. 40042930 PMC11984418

[acel70150-bib-0011] Dubey, S. K. , R. Dubey , S. C. Prajapati , et al. 2024. “Histone Deficiency and Hypoacetylation in the Aging Retinal Pigment Epithelium.” Aging Cell 23, no. 5: e14108. 10.1111/acel.14108. 38408164 PMC11113634

[acel70150-bib-0012] Elizarraras, J. M. , Y. Liao , Z. Shi , Q. Zhu , A. R. Pico , and B. Zhang . 2024. “WebGestalt 2024: Faster Gene Set Analysis and New Support for Metabolomics and Multi‐Omics.” Nucleic Acids Research 52, no. W1: W415–W421. 10.1093/nar/gkae456. 38808672 PMC11223849

[acel70150-bib-0013] Feng, L. , K. Nie , Q. Huang , and W. Fan . 2022. “Complement Factor H Deficiency Combined With Smoking Promotes Retinal Degeneration in a Novel Mouse Model.” Experimental Biology and Medicine (Maywood, N.J.) 247, no. 2: 77–86. 10.1177/15353702211052245. PMC8777480 34775843

[acel70150-bib-0014] Fleckenstein, M. , S. Schmitz‐Valckenberg , and U. Chakravarthy . 2024. “Age‐Related Macular Degeneration: A Review.” JAMA 331, no. 2: 147–157. 10.1001/jama.2023.26074. 38193957 PMC12935482

[acel70150-bib-0015] Fujihara, M. , N. Nagai , T. E. Sussan , S. Biswal , and J. T. Handa . 2008. “Chronic Cigarette Smoke Causes Oxidative Damage and Apoptosis to Retinal Pigmented Epithelial Cells in Mice.” PLoS One 3, no. 9: e3119. 10.1371/journal.pone.0003119. 18769672 PMC2518621

[acel70150-bib-0016] Galiatsatos, P. , B. Kaplan , D. G. Lansey , and A. Ellison‐Barnes . 2023. “Tobacco Use and Tobacco Dependence Management.” Clinics in Chest Medicine 44, no. 3: 479–488. 10.1016/j.ccm.2023.03.004. 37517828

[acel70150-bib-0017] Hanus, J. , C. Anderson , and S. Wang . 2015. “RPE Necroptosis in Response to Oxidative Stress and in AMD.” Ageing Research Reviews 24: 286–298. 10.1016/j.arr.2015.09.002. 26369358 PMC4661094

[acel70150-bib-0018] He, J. , X. Shangguan , W. Zhou , et al. 2021. “Glucose Limitation Activates AMPK Coupled SENP1‐Sirt3 Signalling in Mitochondria for T Cell Memory Development.” Nature Communications 12, no. 1: 4371. 10.1038/s41467-021-24619-2. PMC8285428 34272364

[acel70150-bib-0019] Hernandez‐Segura, A. , J. Nehme , and M. Demaria . 2018. “Hallmarks of Cellular Senescence.” Trends in Cell Biology 28, no. 6: 436–453. 10.1016/j.tcb.2018.02.001. 29477613

[acel70150-bib-0020] Huang, Y. Q. , J. N. Xu , Y. Huang , et al. 2024. “Independent and Combined Effects of Smoking, Drinking and Depression on Periodontal Disease.” BMC Oral Health 24, no. 1: 535. 10.1186/s12903-024-04287-6. 38711116 PMC11075253

[acel70150-bib-0021] Istrate, M. , M. Hasbei‐Popa , D. A. Iliescu , A. C. Ghita , and A. M. Ghita . 2021. “Effects of Cigarette Smoking on Sensorineural Hearing Impairment and Age Related Macular Degeneration.” Tobacco Prevention and Cessation 7: 55. 10.18332/tpc/138952. 34395952 PMC8328227

[acel70150-bib-0022] Karimi, S. , H. Nouri , S. Mahmoudinejad‐Azar , and S. H. Abtahi . 2023. “Smoking and Environmental Tobacco Smoke Exposure: Implications in Ocular Disorders.” Cutaneous and Ocular Toxicology 42, no. 1: 1–7. 10.1080/15569527.2022.2144874. 36369835

[acel70150-bib-0023] Kaufmann, M. , and Z. Han . 2024. “RPE Melanin and Its Influence on the Progression of AMD.” Ageing Research Reviews 99: 102358. 10.1016/j.arr.2024.102358. 38830546 PMC11260545

[acel70150-bib-0024] Kim, J. , H. Song , J. Lee , et al. 2023. “Smoking and Passive Smoking Increases Mortality Through Mediation Effect of Cadmium Exposure in the United States.” Scientific Reports 13, no. 1: 3878. 10.1038/s41598-023-30988-z. 36890267 PMC9995499

[acel70150-bib-0025] Kuan, V. , A. Warwick , A. Hingorani , et al. 2021. “Association of Smoking, Alcohol Consumption, Blood Pressure, Body Mass Index, and Glycemic Risk Factors With Age‐Related Macular Degeneration: A Mendelian Randomization Study.” JAMA Ophthalmology 139, no. 12: 1299–1306. 10.1001/jamaophthalmol.2021.4601. 34734970 PMC8569599

[acel70150-bib-0026] Kuehnemann, C. , and C. D. Wiley . 2024. “Senescent Cells at the Crossroads of Aging, Disease, and Tissue Homeostasis.” Aging Cell 23, no. 1: e13988. 10.1111/acel.13988. 37731189 PMC10776127

[acel70150-bib-0027] Kulkarni, A. , and S. Banait . 2023. “Through the Smoke: An In‐Depth Review on Cigarette Smoking and Its Impact on Ocular Health.” Cureus 15, no. 10: e47779. 10.7759/cureus.47779. 38021969 PMC10676518

[acel70150-bib-0028] Li, Y. , and S. S. Hecht . 2022. “Carcinogenic Components of Tobacco and Tobacco Smoke: A 2022 Update.” Food and Chemical Toxicology 165: 113179. 10.1016/j.fct.2022.113179. 35643228 PMC9616535

[acel70150-bib-0029] Ma, X. , H. Chen , S. Jian , et al. 2023. “DAPL1 Deficiency in Mice Impairs Antioxidant Defenses in the RPE and Leads to Retinal Degeneration With AMD‐Like Features.” Redox Biology 62: 102675. 10.1016/j.redox.2023.102675. 36933392 PMC10031543

[acel70150-bib-0030] Nakanishi, H. , K. Yamashiro , R. Yamada , et al. 2010. “Joint Effect of Cigarette Smoking and CFH and LOC387715/HTRA1 Polymorphisms on Polypoidal Choroidal Vasculopathy.” Investigative Ophthalmology & Visual Science 51, no. 12: 6183–6187. 10.1167/iovs.09-4948. 20688737

[acel70150-bib-0031] Preuss, R. , J. Angerer , and H. Drexler . 2003. “Naphthalene–An Environmental and Occupational Toxicant.” International Archives of Occupational and Environmental Health 76, no. 8: 556–576. 10.1007/s00420-003-0458-1. 12920524

[acel70150-bib-0032] Sahu, R. , K. Shah , R. Malviya , et al. 2023. “E‐Cigarettes and Associated Health Risks: An Update on Cancer Potential.” Adv Respir Med 91, no. 6: 516–531. 10.3390/arm91060038. 37987300 PMC10660480

[acel70150-bib-0033] Schumacher, B. , J. Pothof , J. Vijg , and J. H. J. Hoeijmakers . 2021. “The Central Role of DNA Damage in the Ageing Process.” Nature 592, no. 7856: 695–703. 10.1038/s41586-021-03307-7. 33911272 PMC9844150

[acel70150-bib-0034] Sun, Q. , D. Yu , J. Fan , et al. 2022. “Healthy Lifestyle and Life Expectancy at Age 30 Years in the Chinese Population: An Observational Study.” Lancet Public Health 7, no. 12: e994–e1004. 10.1016/S2468-2667(22)00110-4. 35926549 PMC7615002

[acel70150-bib-0035] Tang, M. S. , H. W. Lee , M. W. Weng , et al. 2022. “DNA Damage, DNA Repair and Carcinogenicity: Tobacco Smoke Versus Electronic Cigarette Aerosol.” Mutation Research, Reviews in Mutation Research 789: 108409. 10.1016/j.mrrev.2021.108409. 35690412 PMC9208310

[acel70150-bib-0036] Thornton, J. , R. Edwards , P. Mitchell , R. A. Harrison , I. Buchan , and S. P. Kelly . 2005. “Smoking and Age‐Related Macular Degeneration: A Review of Association.” Eye (London, England) 19, no. 9: 935–944. 10.1038/sj.eye.6701978. 16151432

[acel70150-bib-0037] Vu, A. T. , K. M. Taylor , M. R. Holman , Y. S. Ding , B. Hearn , and C. H. Watson . 2015. “Polycyclic Aromatic Hydrocarbons in the Mainstream Smoke of Popular U.S. Cigarettes.” Chemical Research in Toxicology 28, no. 8: 1616–1626. 10.1021/acs.chemrestox.5b00190. 26158771 PMC4540633

[acel70150-bib-0038] Wang, D. , B. Bruyneel , L. Kamelia , S. Wesseling , I. Rietjens , and P. J. Boogaard . 2020. “In Vitro Metabolism of Naphthalene and Its Alkylated Congeners by Human and Rat Liver Microsomes via Alkyl Side Chain or Aromatic Oxidation.” Chemico‐Biological Interactions 315: 108905. 10.1016/j.cbi.2019.108905. 31765606

[acel70150-bib-0039] Wang, L. , K. D. Kaya , S. Kim , et al. 2020. “Retinal Pigment Epithelium Transcriptome Analysis in Chronic Smoking Reveals a Suppressed Innate Immune Response and Activation of Differentiation Pathways.” Free Radical Biology & Medicine 156: 176–189. 10.1016/j.freeradbiomed.2020.06.004. 32634473 PMC7434665

[acel70150-bib-0040] Wang, L. , T. Liu , X. Wang , et al. 2023. “Microglia‐Derived TNF‐α Contributes to RVLM Neuronal Mitochondrial Dysfunction via Blocking the AMPK‐Sirt3 Pathway in Stress‐Induced Hypertension.” Journal of Neuroinflammation 20, no. 1: 137. 10.1186/s12974-023-02818-6. 37264405 PMC10236846

[acel70150-bib-0041] Wang, S. Q. , L. J. Bao , T. Y. Li , and E. Y. Zeng . 2024. “Potential Health Risk of Human Exposure to Tobacco‐Specific Nitrosamines in Second‐Hand and Third‐Hand Smoke.” Journal of Hazardous Materials 480: 136446. 10.1016/j.jhazmat.2024.136446. 39536341

[acel70150-bib-0042] Warner, K. E. , N. L. Benowitz , A. McNeill , and N. A. Rigotti . 2023. “Nicotine e‐Cigarettes as a Tool for Smoking Cessation.” Nature Medicine 29, no. 3: 520–524. 10.1038/s41591-022-02201-7. 36788367

[acel70150-bib-0043] Woodell, A. , and B. Rohrer . 2014. “A Mechanistic Review of Cigarette Smoke and Age‐Related Macular Degeneration.” Advances in Experimental Medicine and Biology 801: 301–307. 10.1007/978-1-4614-3209-8_38. 24664711

[acel70150-bib-0044] Yang, L. , B. Wang , F. Guo , et al. 2022. “FFAR4 Improves the Senescence of Tubular Epithelial Cells by AMPK/SirT3 Signaling in Acute Kidney Injury.” Signal Transduction and Targeted Therapy 7, no. 1: 384. 10.1038/s41392-022-01254-x. 36450712 PMC9712544

[acel70150-bib-0045] Yang, W. , C. Song , M. Gao , S. Wang , H. Yu , and Y. Li . 2022. “Effects of Smoking on the Retina of Patients With Dry Age‐Related Macular Degeneration by Optical Coherence Tomography Angiography.” BMC Ophthalmology 22, no. 1: 315. 10.1186/s12886-022-02525-5. 35869464 PMC9308247

[acel70150-bib-0046] Yun, J. , S. Hansen , O. Morris , et al. 2023. “Senescent Cells Perturb Intestinal Stem Cell Differentiation Through Ptk7 Induced Noncanonical Wnt and YAP Signaling.” Nature Communications 14, no. 1: 156. 10.1038/s41467-022-35487-9. PMC9834240 36631445

[acel70150-bib-0047] Zhang, L. , L. E. Pitcher , M. J. Yousefzadeh , L. J. Niedernhofer , P. D. Robbins , and Y. Zhu . 2022. “Cellular Senescence: A Key Therapeutic Target in Aging and Diseases.” Journal of Clinical Investigation 132, no. 15: 450. 10.1172/jci158450. PMC9337830 35912854

[acel70150-bib-0048] Zhao, Z. , W. Chen , Y. Cheng , J. Li , and Z. Chen . 2023. “ *Burkholderia cepacia* Immobilized Onto rGO as a Biomaterial for the Removal of Naphthalene From Wastewater.” Environmental Research 235: 116663. 10.1016/j.envres.2023.116663. 37451574

[acel70150-bib-0049] Zhu, X. , Z. Chen , L. Wang , et al. 2022. “Direct Conversion of Human Umbilical Cord Mesenchymal Stem Cells Into Retinal Pigment Epithelial Cells for Treatment of Retinal Degeneration.” Cell Death & Disease 13, no. 9: 785. 10.1038/s41419-022-05199-5. 36096985 PMC9468174

[acel70150-bib-0050] Zhu, X. , Z. Fu , K. Dutchak , et al. 2024. “Cotargeting CDK4/6 and BRD4 Promotes Senescence and Ferroptosis Sensitivity in Cancer.” Cancer Research 84, no. 8: 1333–1351. 10.1158/0008-5472.Can-23-1749. 38277141

