# Supplementary material for: Naphthalene Metabolites From Long‐Term Environmental Tobacco Smoke Induce the Aging of Retinal Pigment Epithelium
Source: Aging Cell. 2025 Jun 20;24(9):e70150. doi: 10.1111/acel.70150 (PMC12419860; doi:10.1111/acel.70150)
Supplement: Supplementary file 1 — Figure S1. Diagram of oxygen and carbon dioxide concentrations in a cigarette exposure model box. Change of O2 and CO2 concentration in self‐made smoker. Each collection point is 30 min apart. Figure S2. NA with its metabolites (1,2‐DHN and 1,2‐NQ) increases SASP secretion through the NF‐kB signaling pathway. (A) The expression of IL‐6, IL‐1B, IL‐18, and MCP‐1 in ARPE‐19 cells treated separately with NA and its metabolites (1‐NAP, 2‐NAP, 1,2‐DHN, and 1,2NQ). (B) Western blot analysis of NF‐kB and p‐NF‐kB expression in ARPE‐19 cells treated separately with NA and its metabolites (1‐NAP, 2‐NAP, 1,2‐DHN and 1,2‐NQ). n = 3. (C) Western blot analysis of NF‐kB and p‐NF‐kB expression in ARPE‐19 cells after treatment with NF‐kB inhibitor QNZ. n = 3. (D) The effect of NF‐kB inhibitor QNZ on SASP (IL‐6, IL‐1B, IL‐18, and MCP‐1) mRNA expression in ARPE‐19 cells treated separately with NA and its metabolites (1‐NAP, 2‐NAP, 1,2‐DHN, and 1,2NQ). Data are presented as means ± SD. p value measured by one‐way ANOVA and post hoc Bonferroni’s test. Ns, not significant, NA, 1,2‐DHN, 1,2‐NQ, NC+QNZ, NA+QNZ, 1,2‐DHN+QNZ or 1,2‐NQ+QNZ versus NC *p < 0.05; **p < 0.01; ***p < 0.001; ****p < 0.0001. NA + QNZ versus NA #### p < 0.0001, ### p < 0.001, ## p < 0.01, # p < 0.5. 1,2‐DHN + QNZ versus 1,2‐DHN $$$$ p < 0.0001, $$$ p < 0.001, $$ p < 0.01, $ p < 0.5. 1,2‐NQ + QNZ versus 1,2‐NQ ++++ p < 0.0001, +++ p < 0.001, ++ p < 0.01, + p < 0.5. Figure S3. The inhibitory effect of cigarette exposure on the activity of AMPK/SIRT3 in RPE of rats. Western blot was used to analyze the expressions of AMPK, p‐AMPK, SIRT3, Ac‐P53 (Lys382) and P53 in RBCC of normal group and cigarette exposure group. n = 3. Data are presented as means ± SD. p value measured by one‐way ANOVA and post hoc Bonferroni’s test. Ns, not significant, *p < 0.05; **p < 0.01; ***p < 0.001; ****p < 0.0001. Figure S4. NA with its metabolites (1,2‐DHN and 1,2‐NQ) induces cellular senescence in ARPE‐19 cells through the P53 pathway. Weste [file ACEL-24-e70150-s003.docx]

**
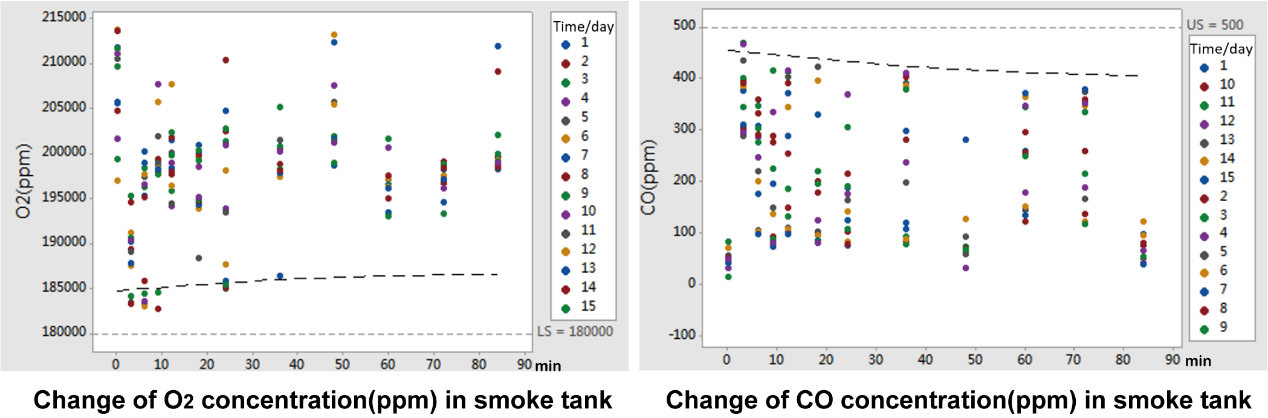
**

**Figure.S1. Diagram of oxygen and carbon dioxide concentrations in a cigarette exposure model box.**

Change of O_2_ and CO_2_ concentration in self-made smoker. Each collection point is 30 minutes apart.


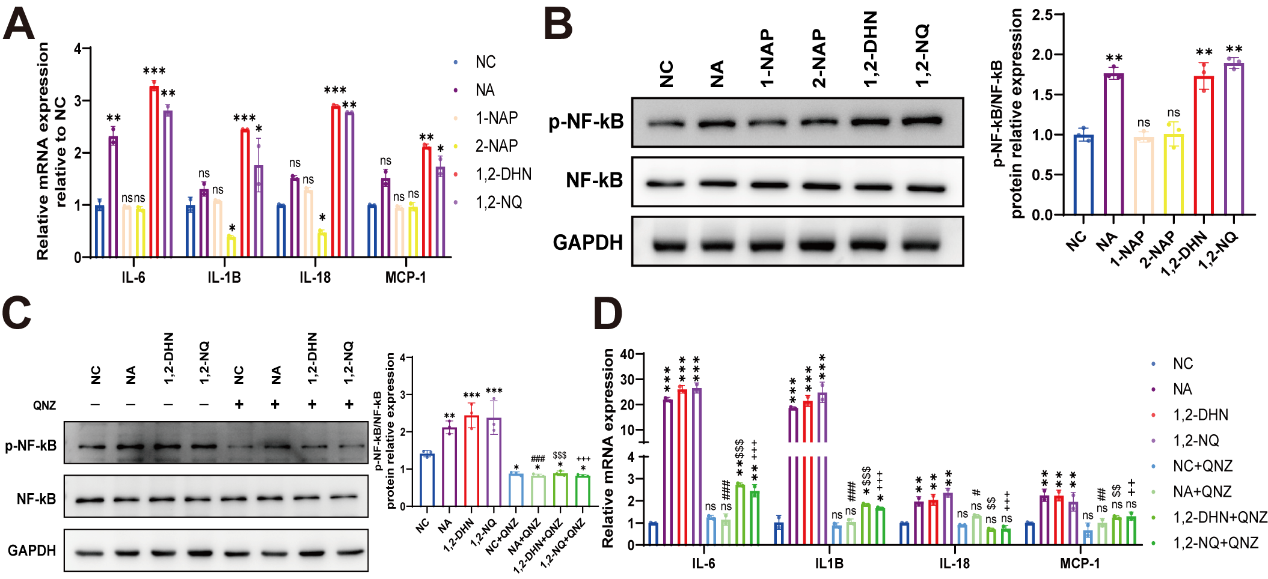


**Supplementary Figure.2. NA with its metabolites (1, 2-DHN and 1, 2-NQ) increases SASP secretion through the NF-kB signaling pathway.**

**(A)** The expression of IL-6, IL-1B, IL-18, and MCP-1 in ARPE-19 cells treated separately with NA and its metabolites (1-NAP, 2-NAP, 1,2-DHN, and 1,2NQ). **(B)** Western blot analysis of NF-kB and p-NF-kB expression in ARPE-19 cells treated separately with NA and its metabolites (1-NAP, 2-NAP, 1,2-DHN and 1,2-NQ). n = 3. **(C)** Western blot analysis of NF-kB and p-NF-kB expression in ARPE-19 cells after treatment with NF-kB inhibitor QNZ. n = 3. **(D)** The effect of NF-kB inhibitor QNZ on SASP (IL-6, IL-1B, IL-18, and MCP-1) mRNA expression in ARPE-19 cells treated separately with NA and its metabolites (1-NAP, 2-NAP, 1,2-DHN, and 1,2NQ). Data are presented as means ± SD. *P* value measured by one-way ANOVA and post hoc Bonferroni’s test. ns, not significant, NA, 1,2-DHN, 1,2-NQ, NC+QNZ, NA+QNZ, 1,2-DHN+QNZ or 1,2-NQ+QNZ vs. NC *P < 0.05; **P < 0.01; ***P < 0.001; ****P < 0.0001. NA +QNZ vs. NA ^####^P < 0.0001, ^###^P < 0.001, ^##^P < 0.01, ^#^P < 0.5. 1,2-DHN +QNZ vs. 1,2-DHN ^$$$$^P < 0.0001, ^$$$^P < 0.001, ^$$^P < 0.01, ^$^P < 0.5. 1,2-NQ +QNZ vs. 1,2-NQ ^++++^P < 0.0001, ^+++^P < 0.001, ^++^P < 0.01, ^+^P < 0.5.


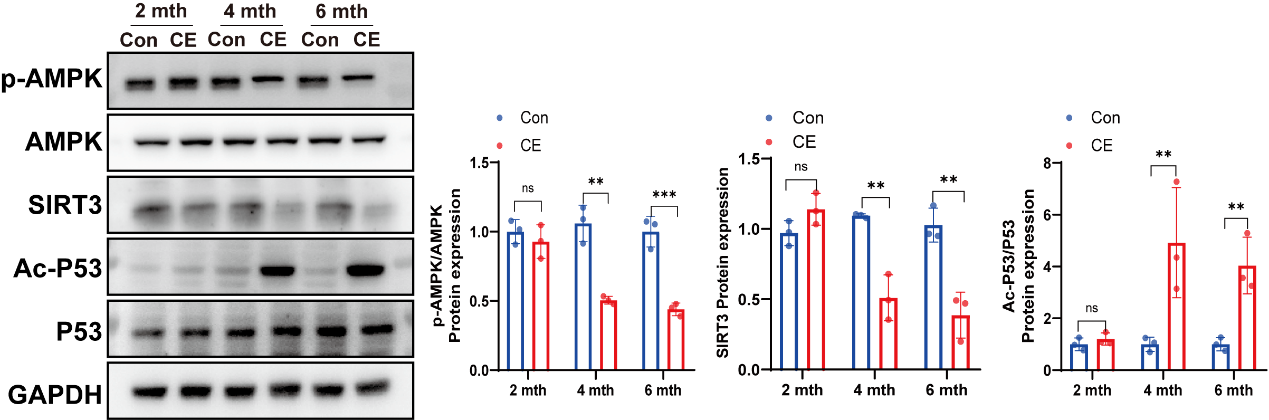


**Supplementary Figure.3. The inhibitory effect of cigarette exposure on the activity of AMPK/SIRT3 in RPE of rats.**

Western blot was used to analyze the expressions of AMPK, p-AMPK, SIRT3, Ac-P53 (Lys382) and P53 in RBCC of normal group and cigarette exposure group. n=3. Data are presented as means ± SD. *P* value measured by one-way ANOVA and post hoc Bonferroni’s test. ns, not significant, *P < 0.05; **P < 0.01; ***P < 0.001; ****P < 0.0001.


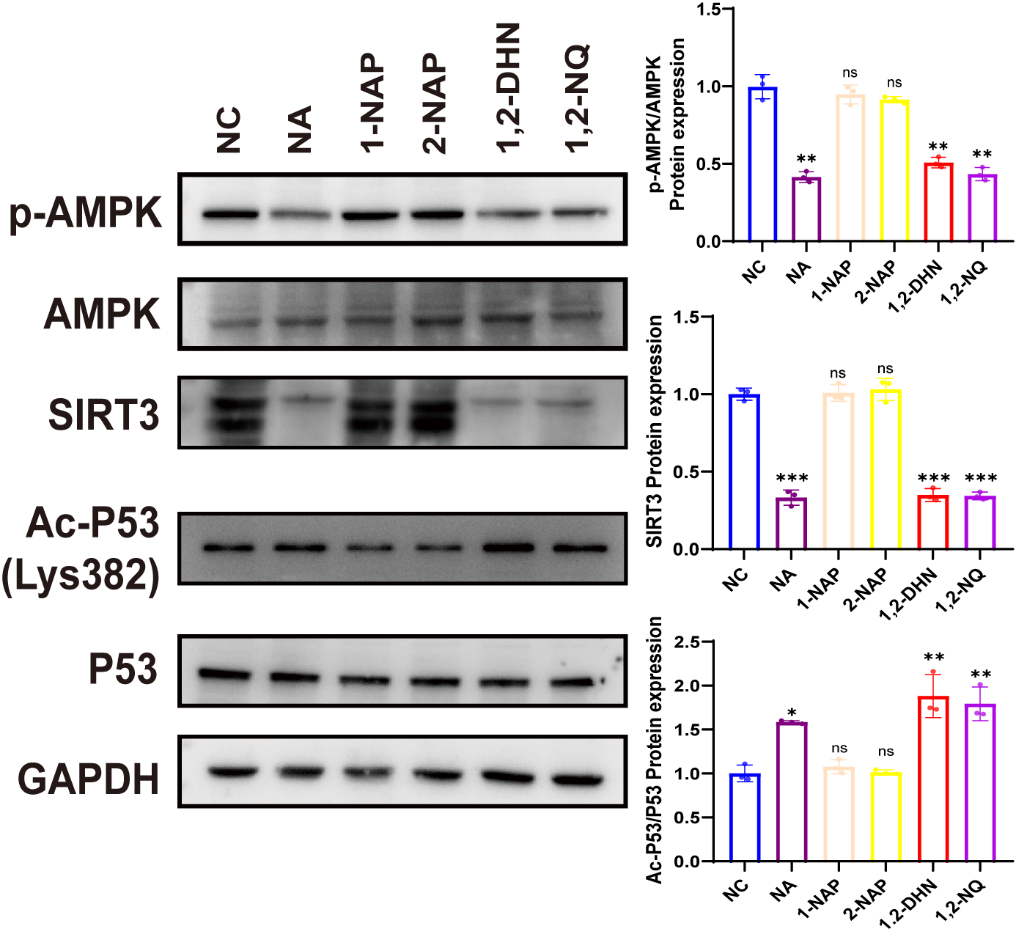


**Supplementary Figure.4. NA with its metabolites (1, 2-DHN and 1, 2-NQ) induces cellular senescence in ARPE-19 cells through the P53 pathway.**

Western blot analysis of AMPK, p-AMPK, SIRT3, Ac-P53 (Lys382) and P53 expression in ARPE-19 cells treated separately with NA and its metabolites (1-NAP, 2-NAP, 1,2-DHN and 1,2NQ). n=3. Data are presented as means ± SD. *P* value measured by one-way ANOVA and post hoc Bonferroni’s test. ns, not significant, *P < 0.05; **P < 0.01; ***P < 0.001; ****P < 0.0001.

**
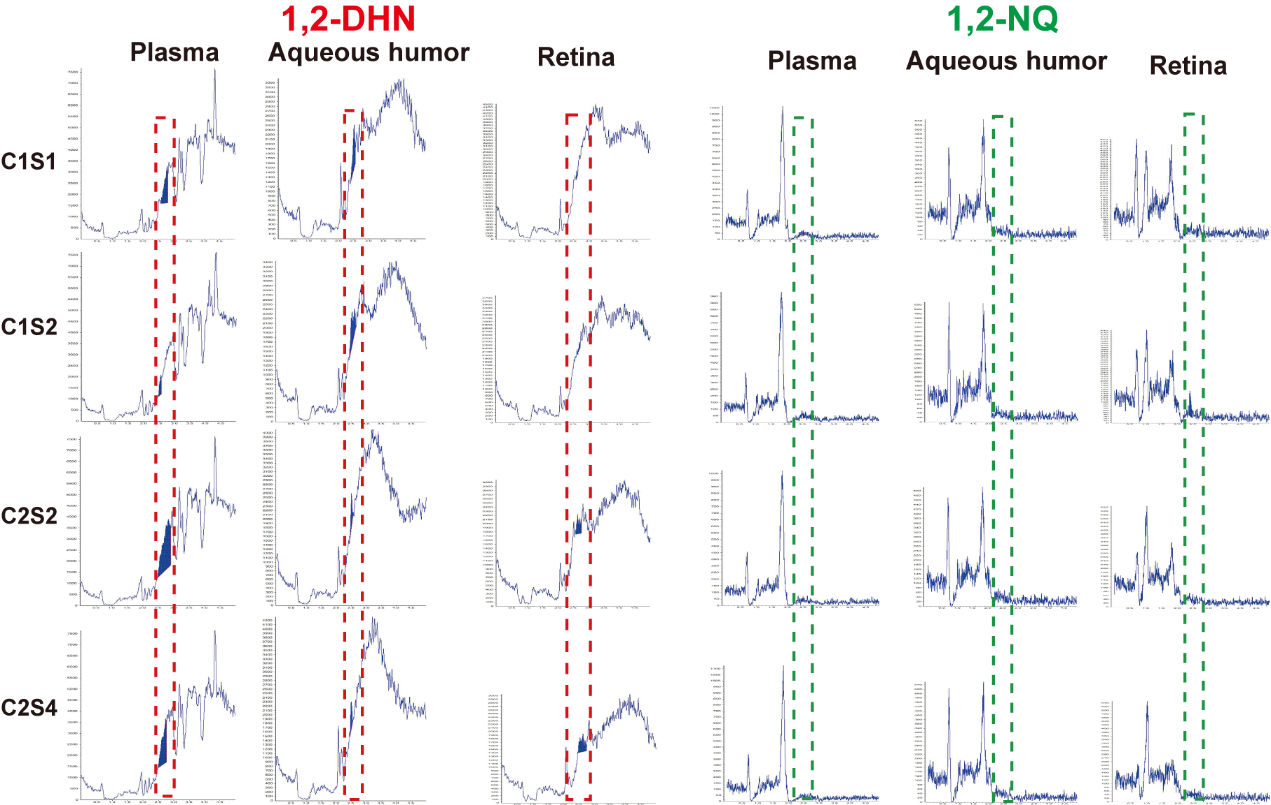
**

**Supplementary Figure.5.** **Smoking cessation can reduce the content of 1, 2-DHN and 1,2-NQ in the aqueous humor of rats.**

Detect 1,2-DHN and 1,2-NQ in plasma , aqueous humor, and retina from smoking cessation rats by LC-MS.


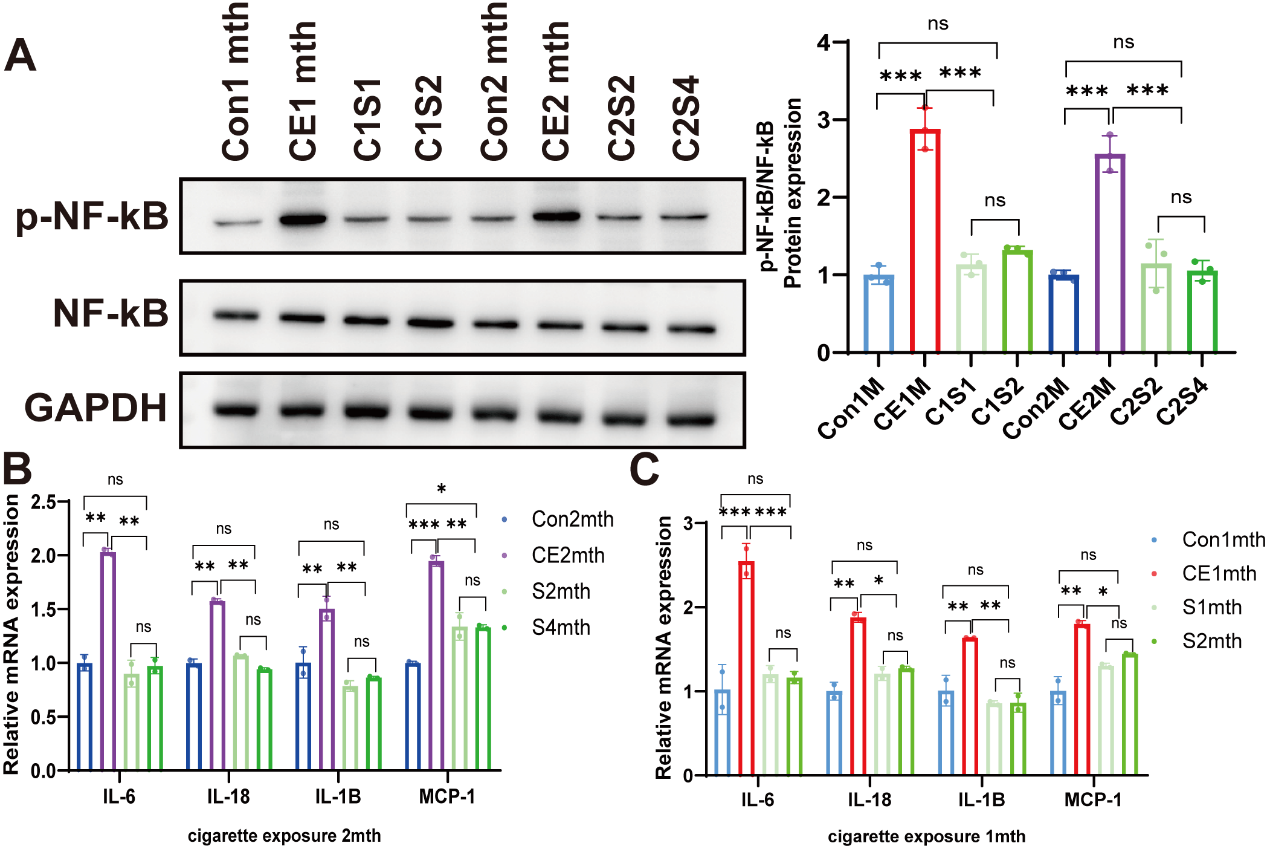


**Supplementary Figure.6.** **Smoking cessation can inhibit the activation of NF-kB and thus reduce the secretion of inflammatory factors.**

**(A)** Western blot analysis and statistics of NF-kB and p-NF-kB expression in ARPE-19 cells. **(B-C)** The expression of SASP (IL-6, IL-1B, IL-18, MCP-1) mRNA levels was detected with qPCR. To ensure experimental robustness, three distinct plasma batches (n=15 per batch) were independently processed and analyzed in all experimental procedures. Data are presented as means ± SD. *P* value measured by one-way ANOVA and post hoc Bonferroni’s test. ns: not significant, *P < 0.05; **P < 0.01; ***P < 0.001; ****P < 0.0001.

**
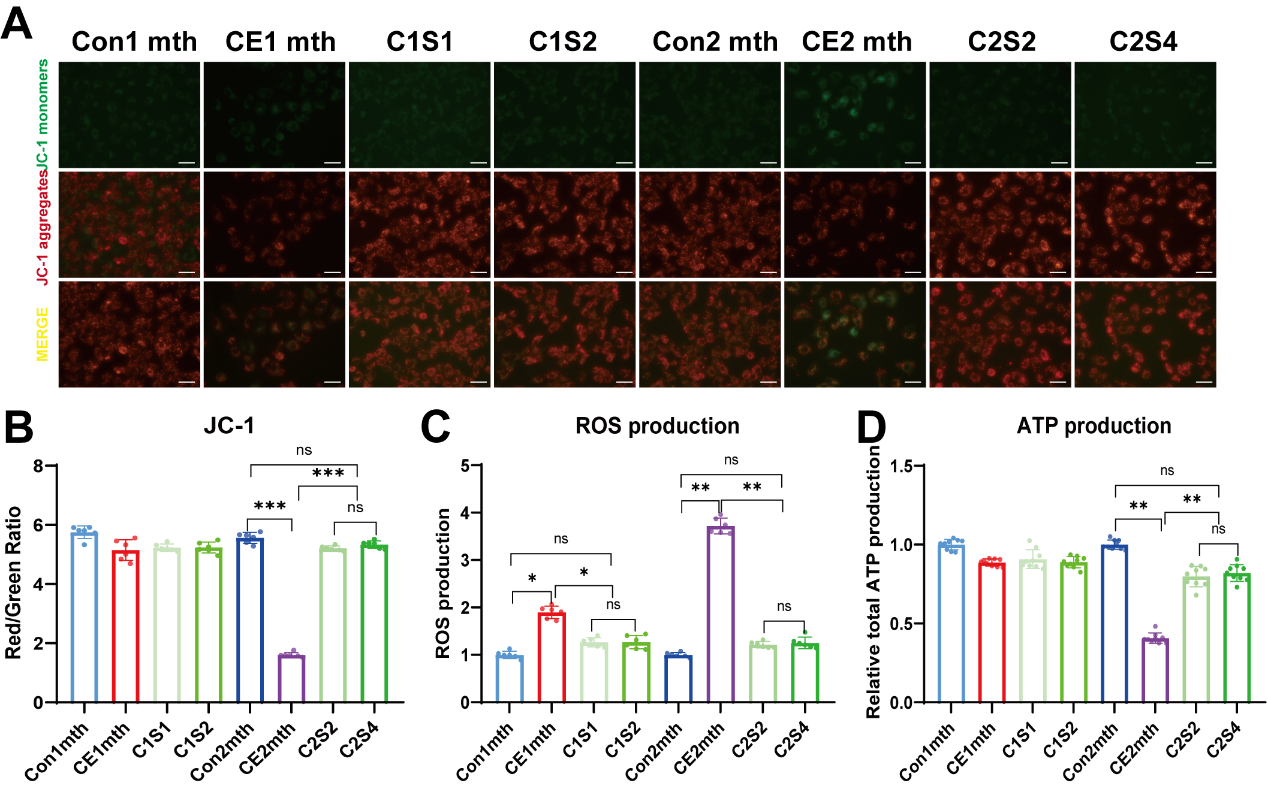
**

**Supplementary Figure.7. Smoking cessation can improve mitochondrial dysfunction caused by cigarette exposure.**

**(A-B)** Immunofluorescence for mitochondrial membrane potential in ARPE-19 cells treated with plasma from a normal group of air-exposed rats (Con), cigarette-exposed rats (CE) and cessation smoking (CS). Scale bar: 20 μm. **(C)** Fluorescence for ROS production in ARPE-19 cells. **(D)** ATP production in ARPE-19 cells. To ensure experimental robustness, three distinct plasma batches (n=15 per batch) were independently processed and analyzed in all experimental procedures. Data are presented as means ± SD. *P* value measured by one-way ANOVA and post hoc Bonferroni’s test. ns, not significant, *P < 0.05; **P < 0.01; ***P < 0.001; ****P < 0.0001.
