# Supplementary material for: Naphthalene Metabolites From Long‐Term Environmental Tobacco Smoke Induce the Aging of Retinal Pigment Epithelium
Source: Aging Cell. 2025 Jun 20;24(9):e70150. doi: 10.1111/acel.70150 (PMC12419860; doi:10.1111/acel.70150)
Supplement: Supplementary file 2 — Table S1. Antibodies used in immunostaining and WB. [file ACEL-24-e70150-s001.docx]

**Supplementary Table 1: Antibodies used in immunostaining and WB.**

| Antibodies | Cat No. | Brand | Dilution ratio |
| --- | --- | --- | --- |
| Acetyl-P53 (Lys382) | #2525 | Cell Signaling Technology | 1:1000 |
| P53 | 21891-1-AP | Proteintech | 1:1000 |
| P38MAPK(Thr180/Tyr182) | #4511 | Cell Signaling Technology | 1:1000 |
| P38 MAPK | 14064-1-AP | Proteintech | 1:1000 |
| P21 | #2947 | Cell Signaling Technology | 1:1000 |
| P16 | 10883-1-AP | Proteintech | 1:1000 |
| Lamin B1  PhosphoHistoneH2A.X(Ser139)  AMPK  Phospho-AMPKα (Thr172)  GAPDH  HRP-conjugated Rabbit Anti-Goat IgG(H+L)  HRP-conjugated Goat Anti-Mouse IgG(H+L) | 12987-1-AP  #9718  10929-2-AP  #2535  HRP-60004  SA00001-4  SA00001-1 | Proteintech  Cell Signaling Technology  Proteintech  Cell Signaling Technology  Proteintech  Proteintech  Proteintech | 1:1000  1:500  1:500  1:1000  1:10000  1:5000  1:5000 |
