# Supplementary material for: Naphthalene Metabolites From Long‐Term Environmental Tobacco Smoke Induce the Aging of Retinal Pigment Epithelium
Source: Aging Cell. 2025 Jun 20;24(9):e70150. doi: 10.1111/acel.70150 (PMC12419860; doi:10.1111/acel.70150)
Supplement: Supplementary file 3 — Table S2. Primers used in RT‐PCR experiments. [file ACEL-24-e70150-s002.docx]

**Supplementary Table 2. Primers used in RT-PCR experiments**

| Primer | Forward (5’-3’) | Reverse (5’-3’) |
| --- | --- | --- |
| Rat-P21  Rat-P16  Rat-IL6  Rat-IL1b  Rat-IL18  Rat-GAPDH  Rat-MCP-1  Human-IL6  Human-IL1b  Human-IL18  Human-MCP-1  Human-GAPDH | GTGTTGACGATGCCTTCTAT  CAGGCATAACTTCTGCTCAA  CTTCTGGAGTTCCGTTTCTAC  AAGTGTCTGAAGCAGCTATG  CCTTCAGAAGCCTGCTATAATC  GAGCATCTCCCTCACAATTC  CTCAGCCAGATGCAGTTAAT  GAGACTTGCCTGGTGAAA  AAGGCGGCCAGGATATAA  TCGGGAAGAGGAAAGGAA  CAGAAGTGGGTTCAGGATTC  CAAGAGCACAAGAGGAAGAG | CCTTGTCCTTGACTTCTTCTT  GAAGTGAAGCCAAGGAGAAA  CTAGGGTTTCAGTATTGCTCTG  CCTCAAAGAACAGGTCATTCT  GCATGGAGCTACAGAGAAAC  GGGTGCAGCGAACTTTAT  GCTGGTGATTCTCTTGTAGTT  TGTTCCTCACTACTCTCAAATC  GGGATTGAGTCCACATTCAG  GGTTCAGCAGCCATCTTTA  GGTTGTGGAGTGAGTGTTC  CTACATGGCAACTGTGAGG |
